# Supplementary material for: Phospholipid composition strongly affects the assembly of β barrel proteins into purified bacterial outer membranes
Source: Nat Commun. 2026 Jan 21;17:1915. doi: 10.1038/s41467-026-68743-3 (PMC12923597; doi:10.1038/s41467-026-68743-3)
Supplement: Supplementary file 1 — Supplementary Information [file 41467_2026_68743_MOESM1_ESM.pdf]

Phospholipid composition strongly affects the assembly of β barrel proteins into purified bacterial outer membranes

Thushani D. Nilaweera[1], Nathan T. Brandes[2], Ian S. LaCroix[2], Benjamin Schwarz[2], and Harris D. Bernstein[1]

This file includes:

Figures S1 to S14

Supplementary References

[1]Genetics and Biochemistry Branch, National Institute of Diabetes and Digestive and Kidney Diseases, National Institutes of Health, Bethesda, MD 20892 USA

[2]Proteins and Chemistry Section, Research and Technologies Branch, Rocky Mountain Laboratories, National Institute of Allergy and Infectious Diseases, National Institutes of Health, Hamilton, MT, USA

Supplementary Figures:

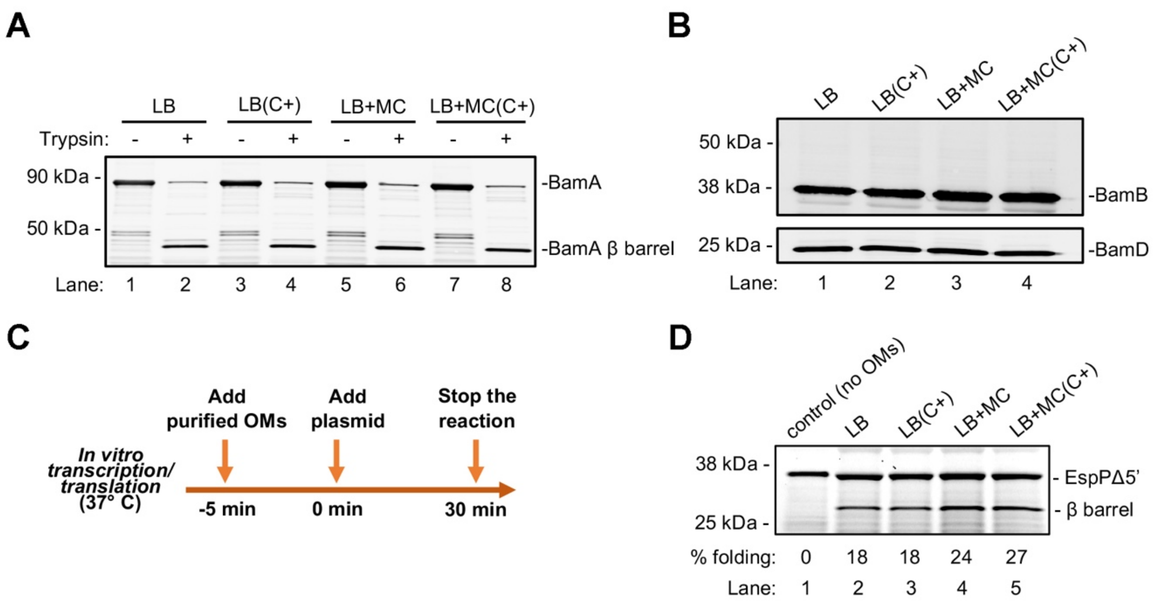

Fig. S1. Characterization of BAM in OMs purified by sarkosyl extraction. MC4100 cells transformed with pJH114 were grown in LB or LB supplemented with $CaCl_2$ and $MgCl_2$ (LB+MC). OMs were purified in 20 mM Tris buffer (pH 8.0) that containing 0.6 mM PMSF and either with 0.6 mM $CaCl_2$ (C+) or no $CaCl_2$. (A) The purified OM samples were incubated in the absence or presence of trypsin (2 mg/mL) for 45 min at 37° C. The levels of BamA and accessible BAM were assessed by Western blotting using an antiserum raised against a C-terminal BamA peptide. (B) The levels of BamB and BamD were monitored by Western blotting using rabbit polyclonal antisera raised against the two proteins. (C) A schematic showing the original experimental design of the EspPΔ5' folding assay using the PURExpress coupled transcription/translation system in the presence of 2 μM SurA and native OMs (containing 2 μM BAM) purified by sarkosyl extraction from MC4100 after BAM expression was induced. (D) At the end of the folding assay samples were heated and the proteins were resolved by SDS-PAGE. Fluorescently labeled EspPΔ5' and the EspPΔ5' β barrel that was generated by self-cleavage after the protein was assembled were visualized at an excitation wavelength of 488 nm. This experiment was repeated eight times with similar results.

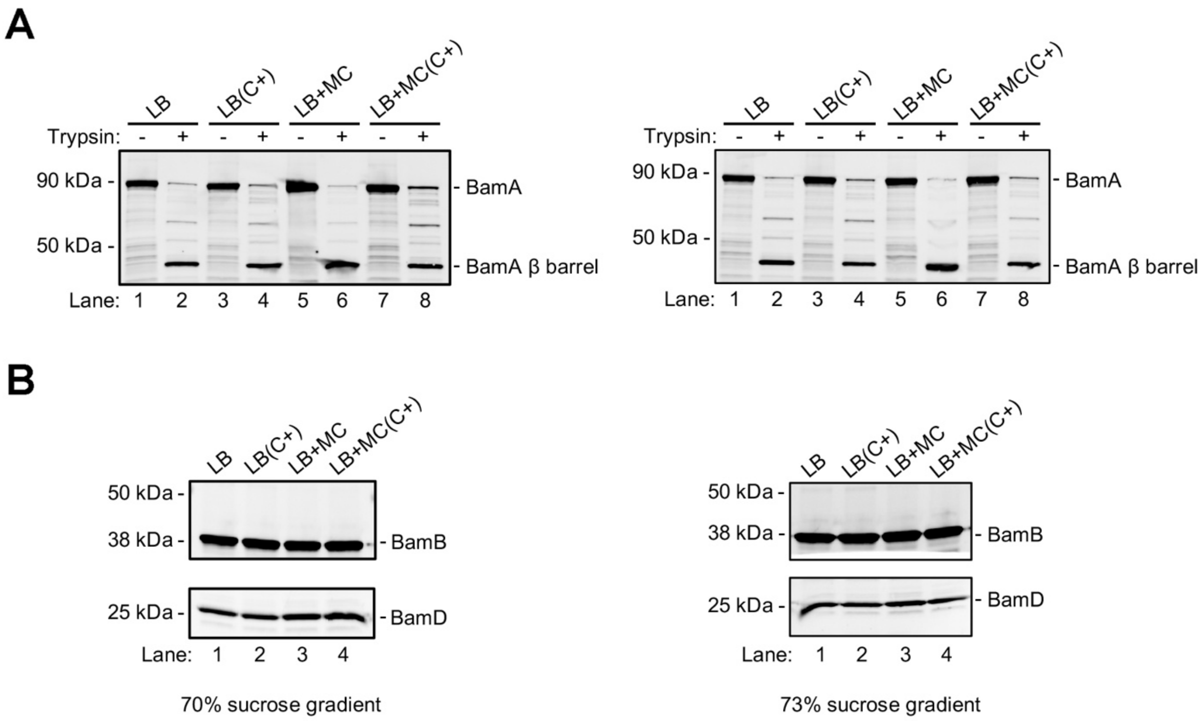

Fig. S2. Characterization of BAM in OMs purified by the sucrose gradient method. MC4100 transformed with pJH114 were grown and processed as in Fig S1. (A) OM samples purified using a 70% sucrose gradient (left) and a 73% sucrose gradient (right) were incubated in the absence or presence of trypsin (2 mg/mL) for 45 min at 37° C. The levels of accessible BamA were assessed by Western blotting using a rabbit polyclonal antiserum raised against a C-terminal BamA peptide. (B) The levels of BamB and BamD in OM samples purified using a 70% sucrose gradient (left) and a 73% sucrose gradient (right) were monitored by Western blotting using rabbit polyclonal antisera raised against the two proteins.

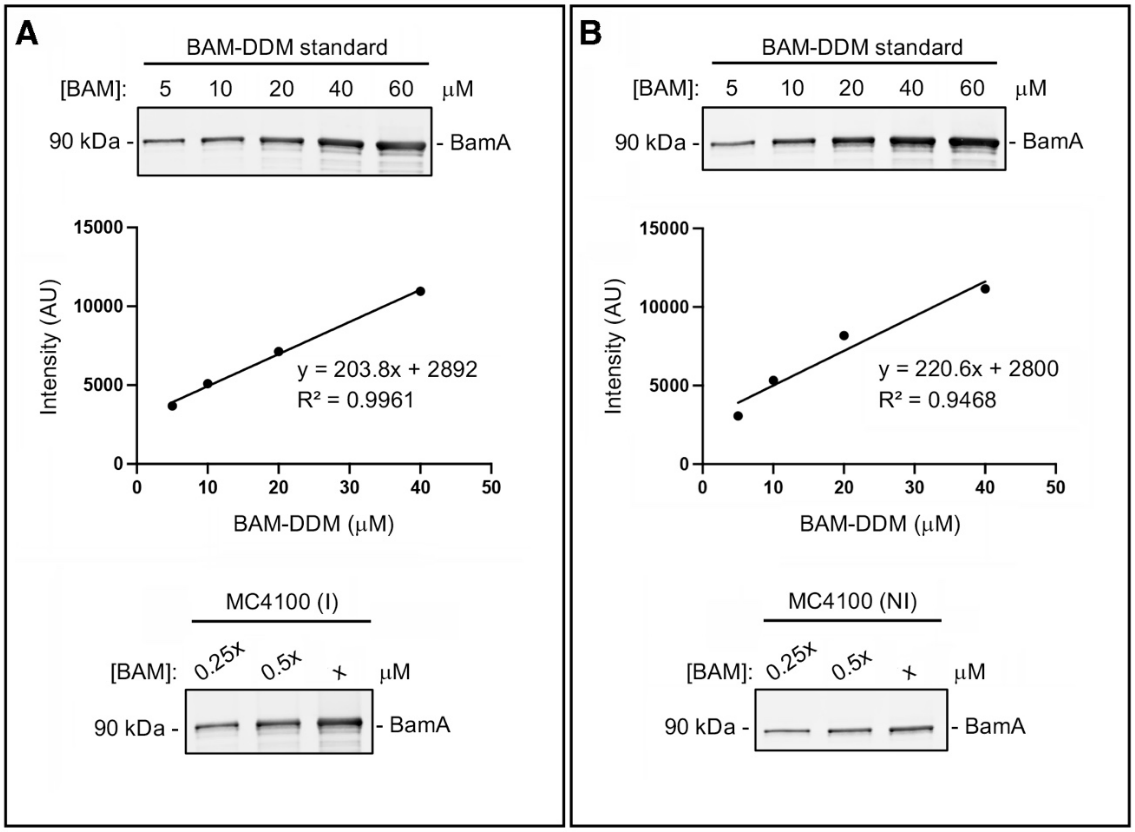

Fig. S3. Quantitation of BAM in native OMs. The concentration of BAM purified in DDM (BAM-DDM) was determined at A_{280} using ε = 294,630 M^{-1} cm^{-1} as previously described[1,2]. Five different amounts of BAM-DDM (5-60 μM) and 0.25x-1x native OMs obtained from MC4100 in which BAM expression was induced [MC4100 (I); A] or non-induced [MC4100 (NI); B] were subjected to SDS-PAGE on two parallel gels and BamA was visualized by Western blot. Blots were analyzed by ImageJ to determine the signal intensity (in arbitrary units, AU) of each concentration of the BAM-DDM standards and the values were plotted using GraphPad Prism (version 10.2.3). Equations for the best fit values that were derived from a simple linear regression model were then used to calculate the BAM concentration in the native OM samples based on the intensity of the BamA signals.

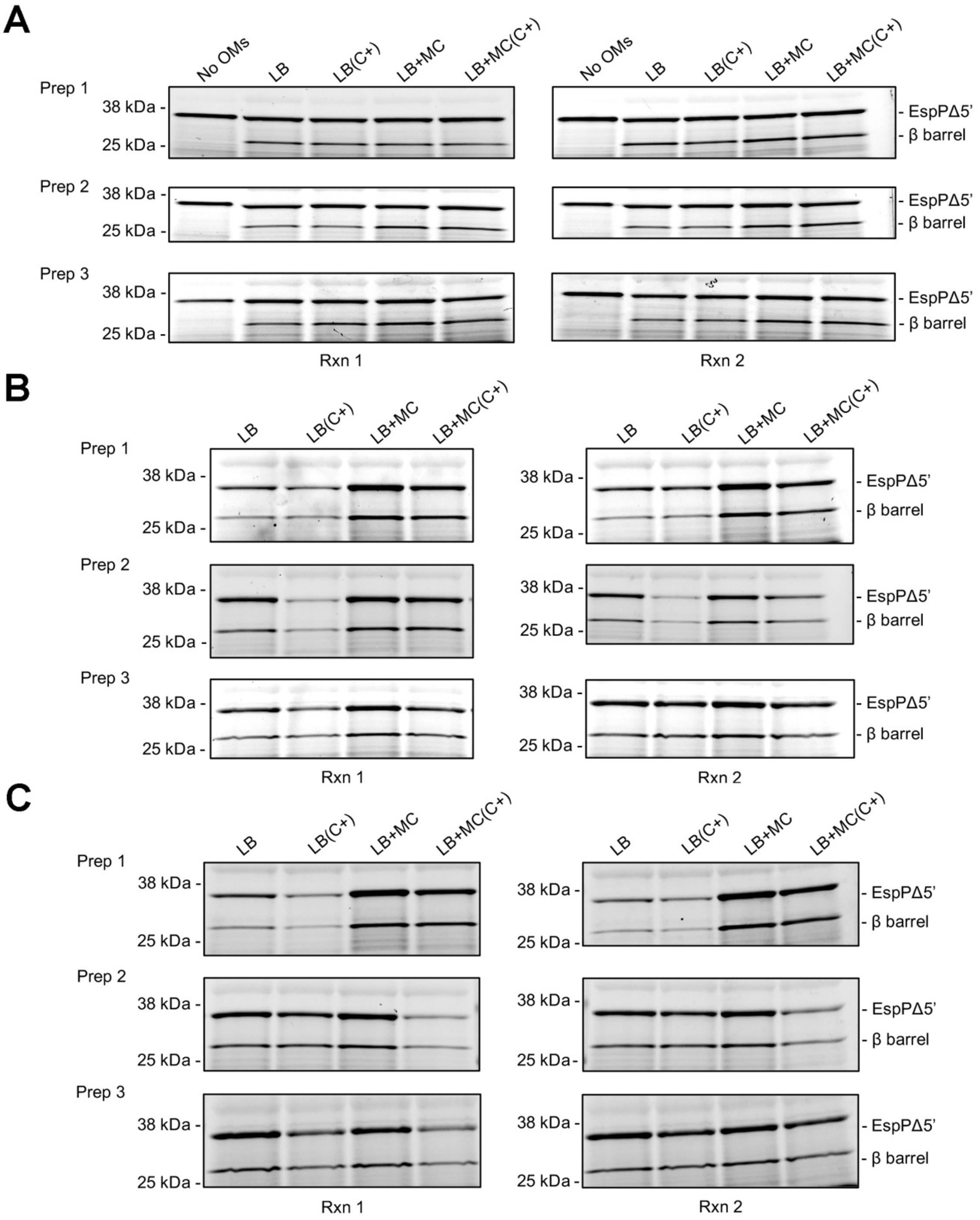

Fig. S4. The assembly of de novo synthesized EspPΔ5' into native OMs purified by sarkosyl extraction is highly reproducible. The experiment shown in Fig. S1D was repeated using three different preparations of native OMs purified from MC4100 by (A) sarkosyl extraction, (B) 70% sucrose gradient fractionation and (C) 73% sucrose gradient fractionation. Each OM preparation was used in two independent trials. At the end of the folding assay samples were heated and proteins were resolved by SDS-PAGE and visualized as in Fig. S1D.

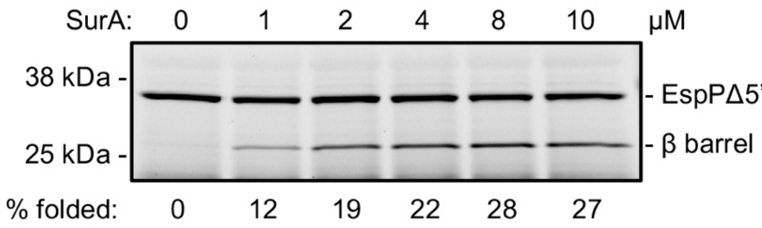

Fig. S5. The effect of varying the concentration of SurA on the assembly of de novo synthesized EspPΔ5'. The experimental scheme illustrated in Fig. 2B was used to monitor the assembly of de novo synthesized EspPΔ5' into native OMs (containing 1 μM BAM) purified from MC4100 after BAM expression was induced in the presence of a variable concentration of SurA (0-10 μM). Samples were incubated for 30 min at 37° C and the fluorescently labeled EspPΔ5' was detected as in Fig. S1D.

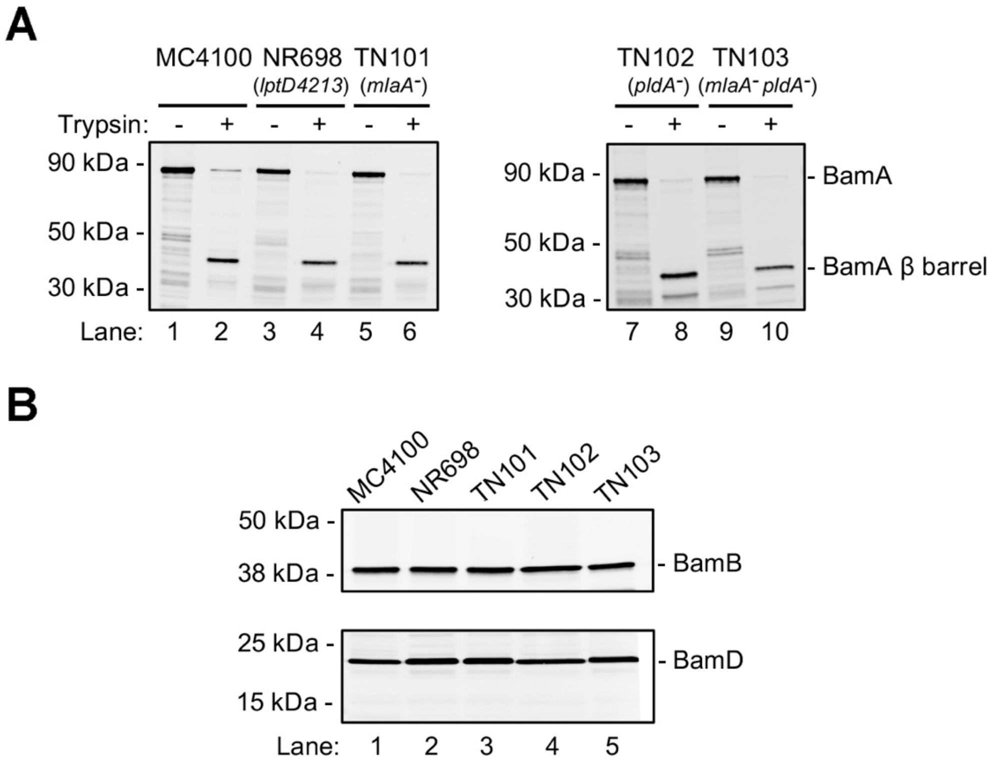

Fig. S6. Characterization of BAM in native OMs purified by sarkosyl extraction from MC4100, NR698, TN101, TN102 and TN103. Wild-type MC4100 and mutant derivatives transformed with pJH114 were grown in LB+MC and native OMs were purified by sarkosyl extraction. (A) The experiment described in Fig. S1A was repeated using OMs purified from the indicated strains. (B) The experiment described in Fig. S1B was repeated using OMs purified from the indicated strains.

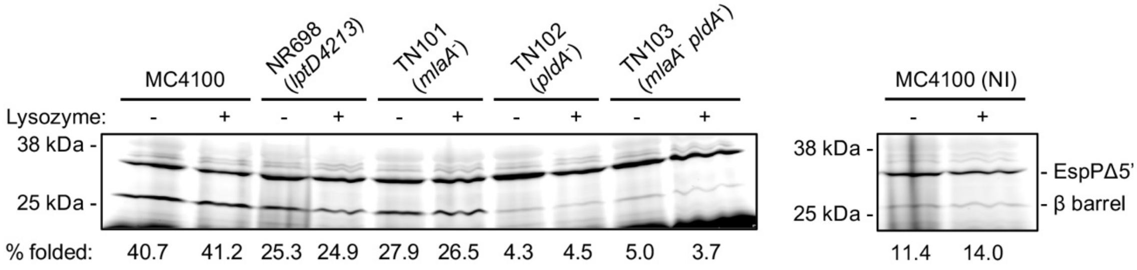

Fig. S7. Residual peptidoglycan on native OMs does not affect the assembly of de novo synthesized EspPΔ5'. The assembly of de novo synthesized EspPΔ5' in the presence of 8 μM SurA and native OMs purified from the indicated strain in which BAM expression was induced (left) or BAM expression was not induced (NI, right). Pre-sonicated OMs containing 1 μM BAM were pre-incubated with or without lysozyme (1 mg/mL) for 10 min on ice. The experimental scheme shown in Fig. 2B was used and samples were collected after a 30 min incubation at 37° C. Following SDS-PAGE fluorescently labeled proteins were detected as in Fig. S1D.

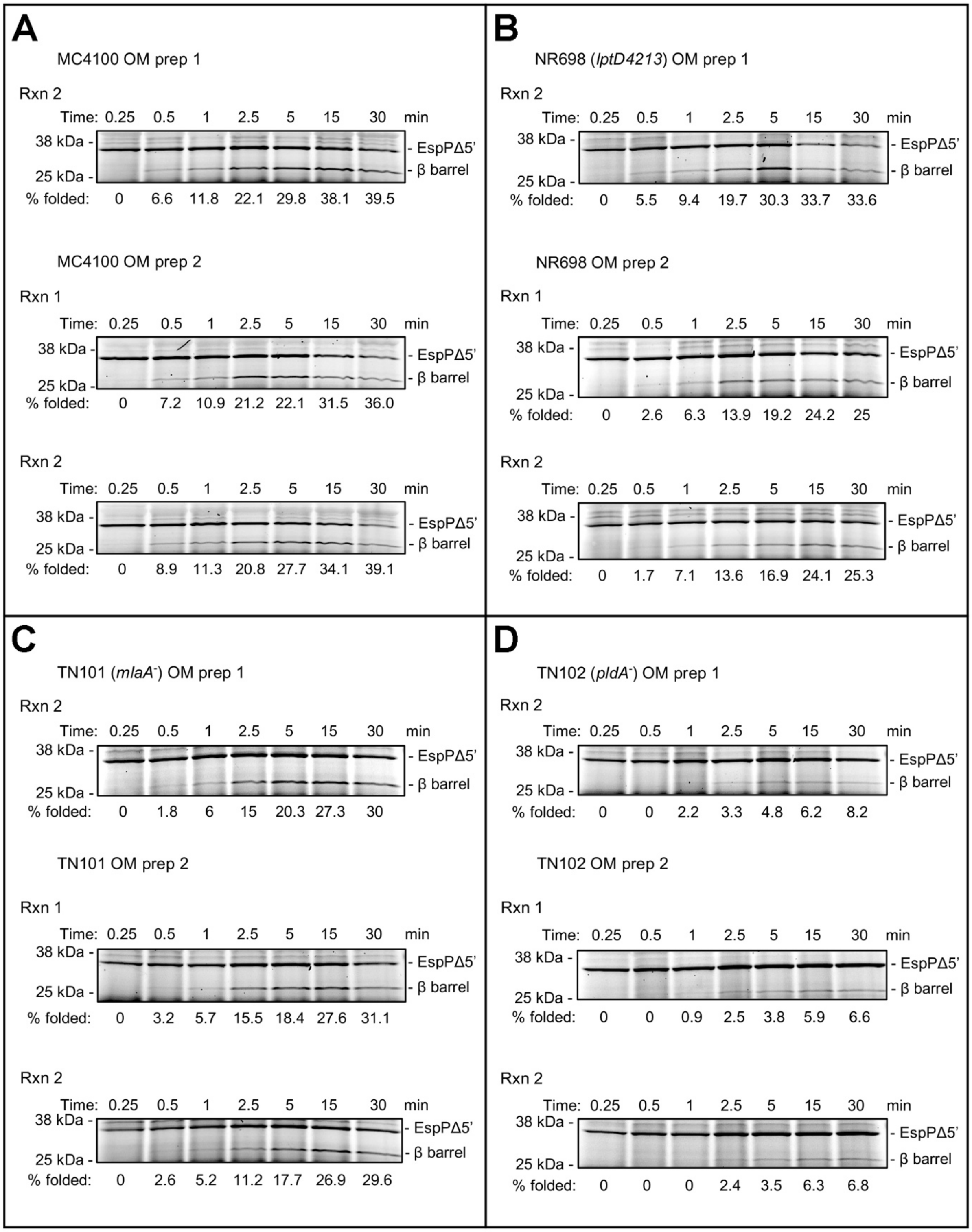
A
MC4100 OM prep 1
Rxn 2
Time: 0.25 0.5 1 2.5 5 15 30 min
38 kDa -
25 kDa -
- EspPΔ5'
- β barrel
% folded: 0 6.6 11.8 22.1 29.8 38.1 39.5
MC4100 OM prep 2
Rxn 1
Time: 0.25 0.5 1 2.5 5 15 30 min
% folded: 0 7.2 10.9 21.2 22.1 31.5 36.0
Rxn 2
Time: 0.25 0.5 1 2.5 5 15 30 min
% folded: 0 8.9 11.3 20.8 27.7 34.1 39.1
B
NR698 (lptD4213) OM prep 1
Rxn 2
Time: 0.25 0.5 1 2.5 5 15 30 min
% folded: 0 5.5 9.4 19.7 30.3 33.7 33.6
NR698 OM prep 2
Rxn 1
Time: 0.25 0.5 1 2.5 5 15 30 min
% folded: 0 2.6 6.3 13.9 19.2 24.2 25
Rxn 2
Time: 0.25 0.5 1 2.5 5 15 30 min
% folded: 0 1.7 7.1 13.6 16.9 24.1 25.3
C
TN101 (mlaA-) OM prep 1
Rxn 2
Time: 0.25 0.5 1 2.5 5 15 30 min
% folded: 0 1.8 6 15 20.3 27.3 30
TN101 OM prep 2
Rxn 1
Time: 0.25 0.5 1 2.5 5 15 30 min
% folded: 0 3.2 5.7 15.5 18.4 27.6 31.1
Rxn 2
Time: 0.25 0.5 1 2.5 5 15 30 min
% folded: 0 2.6 5.2 11.2 17.7 26.9 29.6
D
TN102 (pldA-) OM prep 1
Rxn 2
Time: 0.25 0.5 1 2.5 5 15 30 min
% folded: 0 0 2.2 3.3 4.8 6.2 8.2
TN102 OM prep 2
Rxn 1
Time: 0.25 0.5 1 2.5 5 15 30 min
% folded: 0 0 0.9 2.5 3.8 5.9 6.6
Rxn 2
Time: 0.25 0.5 1 2.5 5 15 30 min
% folded: 0 0 0 2.4 3.5 6.3 6.8

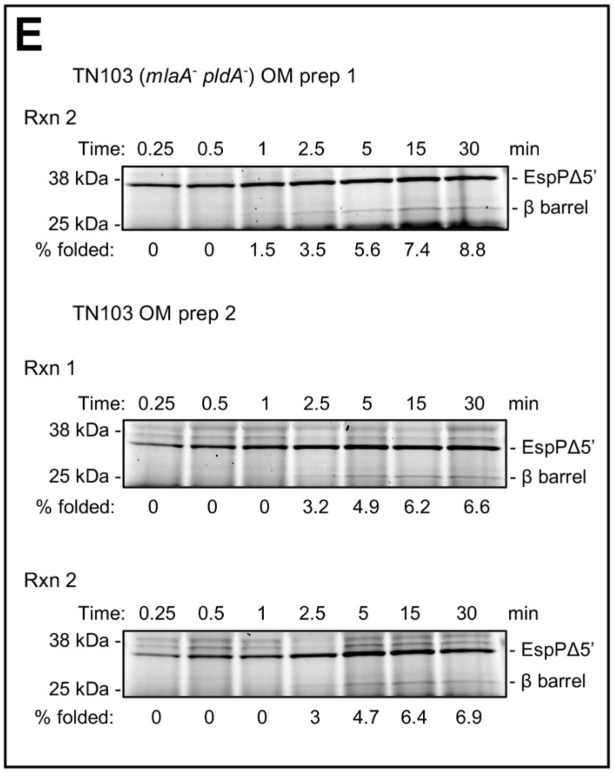

Fig. S8. Assembly assay results used for Fig. 4. The experiment shown in Figs. 4A, right and 4B was repeated three additional times using native OMs purified from (A) MC4100, (B) NR698 (*lptD*4213), (C) TN101 (*mlaA*$^-$), (D) TN102 (*pldA*$^-$) and (E) TN103 (*mlaA*$^-$ *pldA*$^-$) cells and the data from all four experiments was used to construct the plot in Fig. 4C.

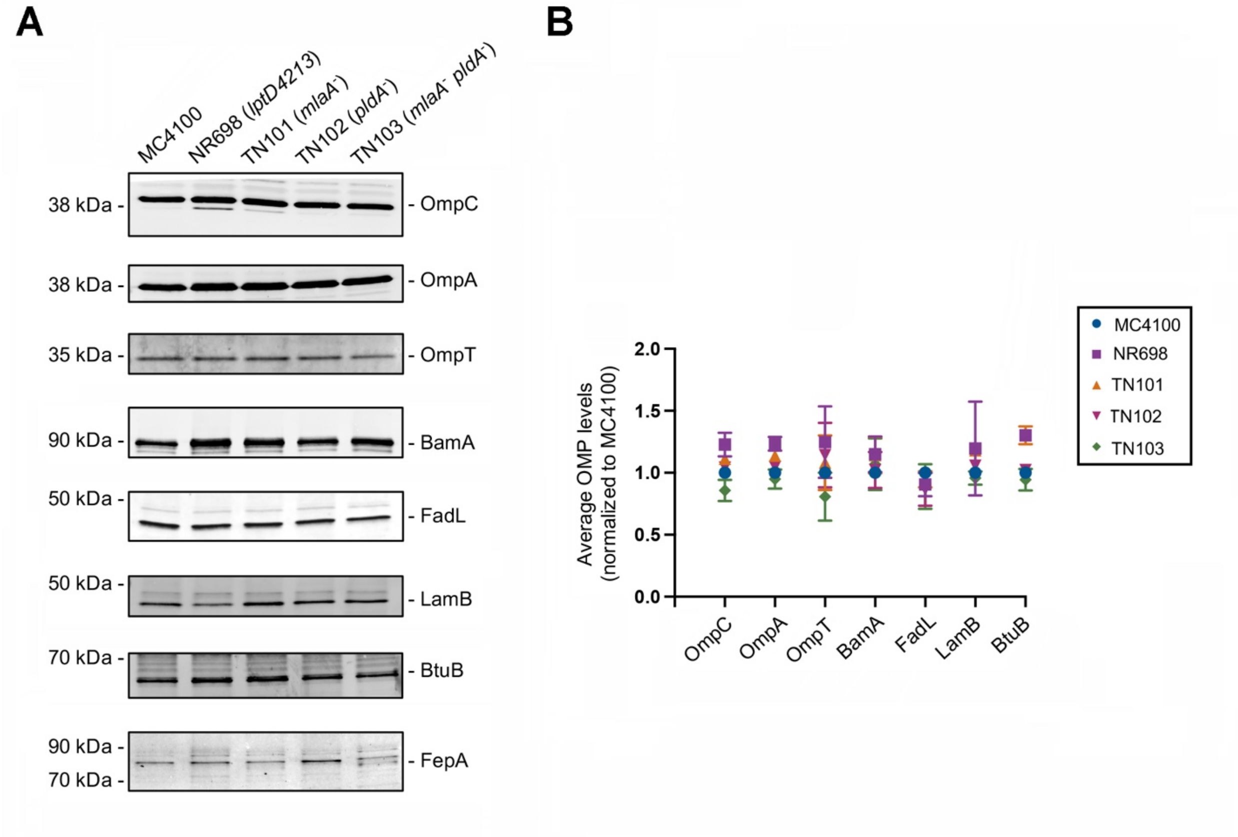

Fig. S9. The levels of model OMPs in MC4100, NR698, TN101, TN102 and TN103 at late log phase. (A) MC4100 and the indicated mutant strains transformed with pJH114 were grown overnight in LB+MC, diluted, and regrown to OD_{600} ~ 0.8, at which point BAM expression was induced by adding 0.4 mM IPTG for 45 min. 0.04 OD_{600} equivalents were then heated and proteins were resolved by SDS-PAGE. OMPs were detected by Western blot using rabbit polyclonal antisera raised against the indicated proteins. Blots from one of the three replicates are shown. (B) The signal intensities of OMPs from NR698, TN101, TN102 and TN103 detected in all three replicates were normalized to the corresponding OMP signal intensities in the MC4100 samples. The average values of normalized signals (where MC4100 = 1.0) are shown in the plot. The error bars represent the standard error. FepA was excluded from the analysis because the signal intensities were too close to the background signal to obtain accurate values.

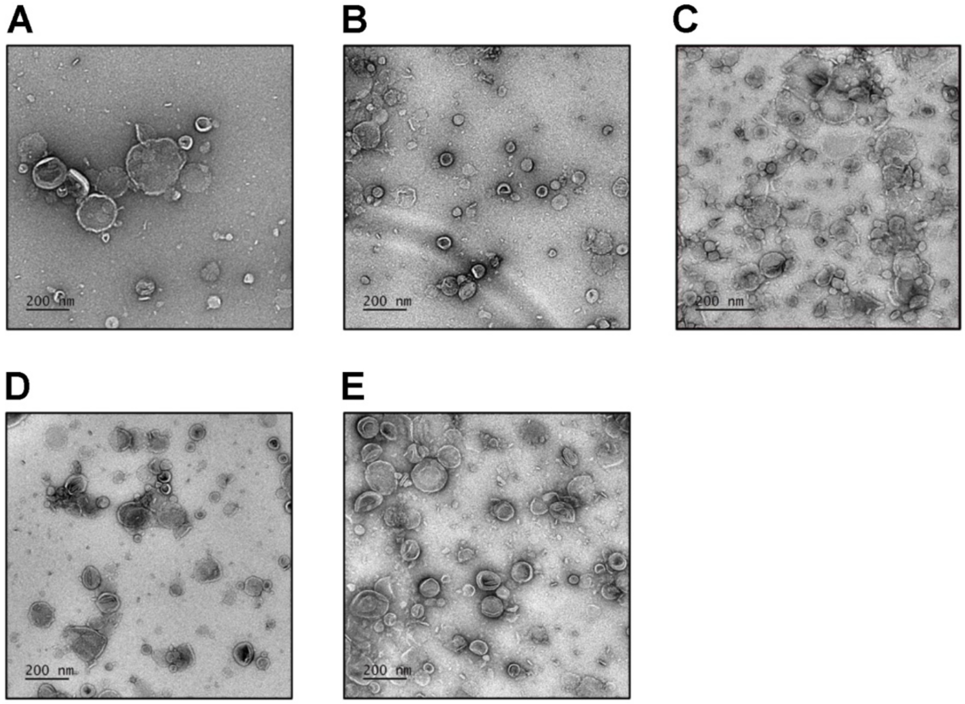

Fig. S10. TEM images of purified native OMs. OM samples from (A) MC4100, (B) NR698 (*lptD*4213), (C) TN101(*mlaA*$^-$), (D) TN102 (*pldA*$^-$) and (E) TN103 (*mlaA*$^-$ *pldA*$^-$) cells were visualized using a FEI Tecnai T12 microscope. Both intact vesicles and fragments of ruptured vesicles were detected in each purified OM sample. A scale bar of 200 nm is shown.

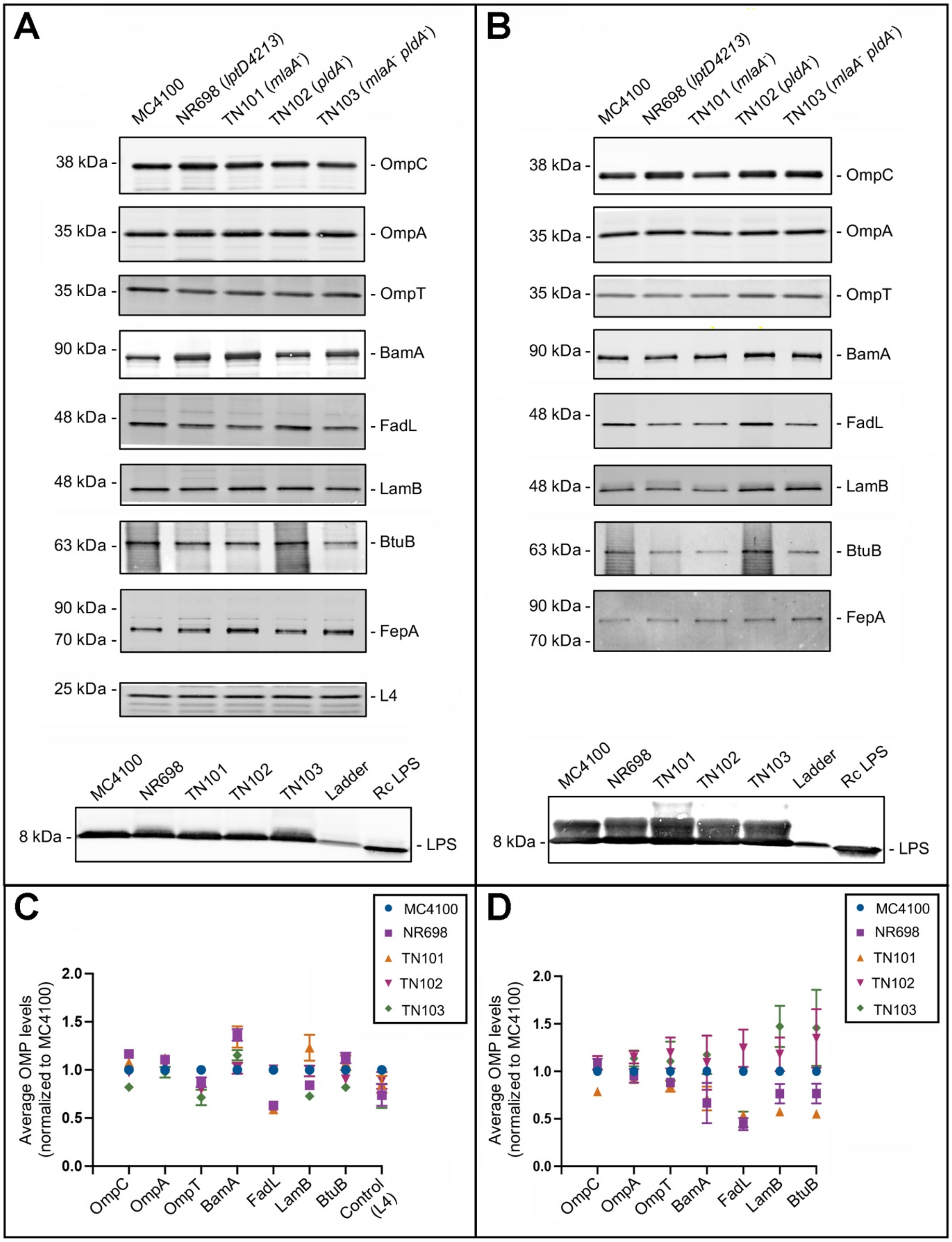
A
MC4100
NR698 (lptD4213)
TN101 (mlaA-)
TN102 (pldA-)
TN103 (mlaA- pldA-)
38 kDa - OmpC
35 kDa - OmpA
35 kDa - OmpT
90 kDa - BamA
48 kDa - FadL
48 kDa - LamB
63 kDa - BtuB
90 kDa - 70 kDa - FepA
25 kDa - L4
MC4100 NR698 TN101 TN102 TN103 Ladder Rc LPS
8 kDa - LPS
B
MC4100
NR698 (lptD4213)
TN101 (mlaA-)
TN102 (pldA-)
TN103 (mlaA- pldA-)
38 kDa - OmpC
35 kDa - OmpA
35 kDa - OmpT
90 kDa - BamA
48 kDa - FadL
48 kDa - LamB
63 kDa - BtuB
90 kDa - 70 kDa - FepA
MC4100 NR698 TN101 TN102 TN103 Ladder Rc LPS
8 kDa - LPS
C
MC4100
NR698
TN101
TN102
TN103
Average OMP levels (normalized to MC4100)
2.0
1.5
1.0
0.5
0.0
OmpC OmpA OmpT BamA FadL LamB BtuB Control (L4)
D
MC4100
NR698
TN101
TN102
TN103
Average OMP levels (normalized to MC4100)
2.0
1.5
1.0
0.5
0.0
OmpC OmpA OmpT BamA FadL LamB BtuB

Fig. S11. The levels of model OMPs and LPS in WC samples and native OMs purified from MC4100, NR698, TN101, TN102 and TN103 that were used in the lipidomics analysis. MC4100 and the indicated mutant strains transformed with pJH114 were grown to OD_{600} = 0.8, at which point BAM expression was induced for 45 min by adding 0.4 M IPTG. WC samples from each culture were collected prior to the purification of native OMs using the sarkosyl extraction method. (A) 0.1 OD_{600} equivalents from WCs and (B) 100 x diluted purified OM samples (0.12-0.14 OD_{600} equivalents from the original culture) that were used in the lipidomics study were then heated and subjected to SDS-PAGE. OMPs were detected by Western blotting using rabbit polyclonal antisera raised against the indicated OMPs while LPS was detected by Western blotting using a mouse monoclonal antibody raised against *E. coli* LPS. Blots from one of the four replicates are shown. The signal intensities of OMPs from NR698, TN101, TN102 and TN103 detected by Western blot in all four replicates were normalized to the corresponding signal intensities of MC4100 in the (C) WC and (D) purified native OM samples. The average values of the normalized signals are shown in the plot. The ribosomal L4 subunit was used as an internal control in the analysis of WC samples. The error bars represent the standard error. FepA was excluded from the analysis because the signal intensities in the purified OM samples were too close to the background signal to obtain accurate values.

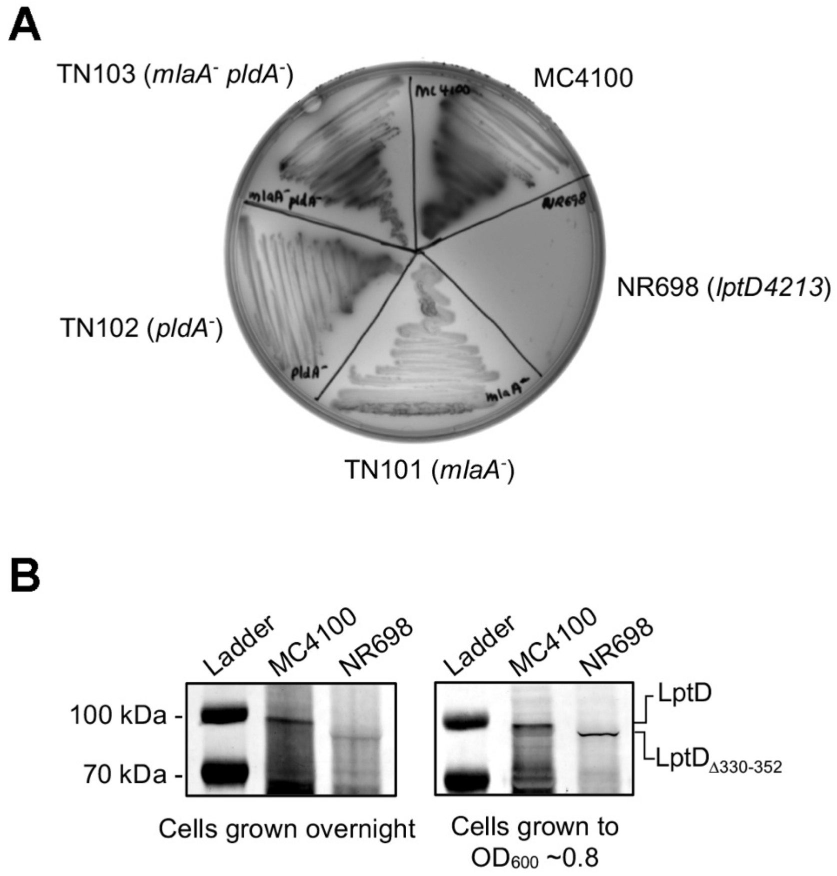

Fig. S12. Analysis of NR698 phenotype. (A) MC4100 and the indicated mutant strains transformed with pJH114 were streaked on violet red bile glucose agar (VRBGA) and incubated overnight at 37° C. Consistent with previous results[3,4], NR698 was sensitive to bile salts and did not grow. (B) MC4100 and NR698 containing pJH114 were grown in LB overnight, diluted, and grown to OD_{600} ~0.8. Cell pellets were resuspended in BugBuster® master mix, incubated at room temperature for 15 min, and then mixed with DTT (25 mM) and 2x SDS-PAGE loading buffer. Samples were heated and OD_{600}= 2.5 and 0.8 equivalents from overnight and log phase cultures, respectively, were subjected to SDS-PAGE. LptD was detected by Western blotting using a rabbit polyclonal antiserum raised against LptD. The results confirm that NR698 harbors the *imp4213* mutation that produces a 23 amino acid deletion in LptD.

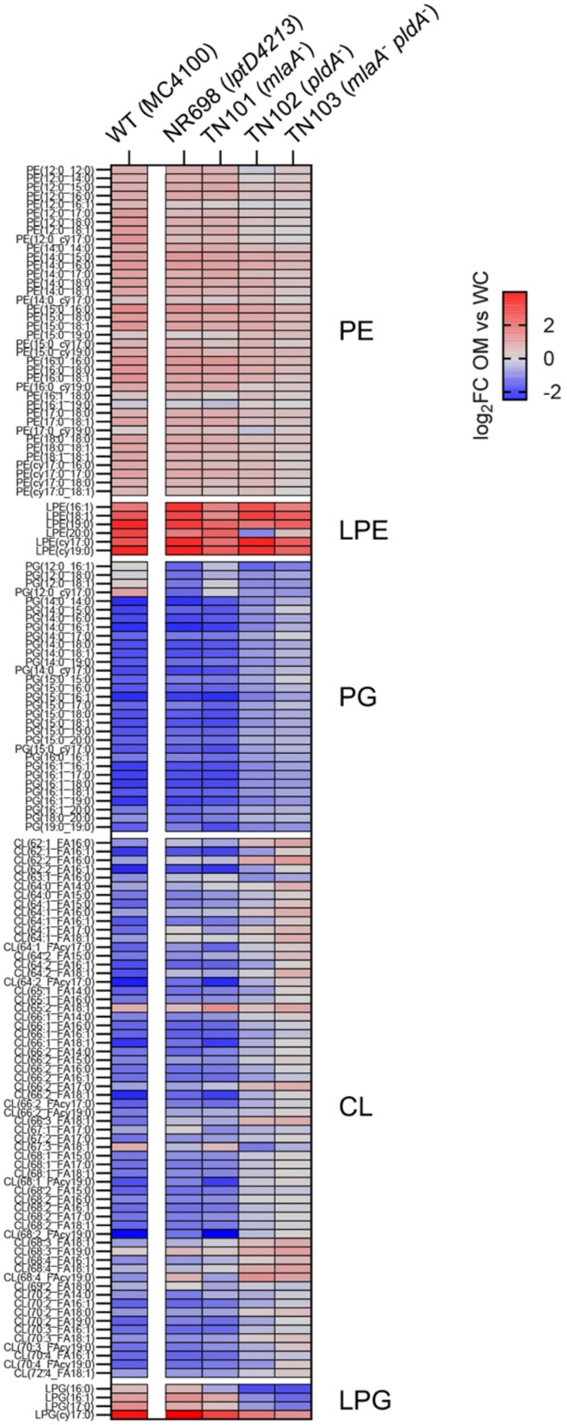

Fig. S13. Expanded heatmap showing OM to WC PL differential in MC4100 and mutant strains. After inducing BAM expression with IPTG, WC samples from each *E. coli* strain were collected prior to the purification of native OMs using the sarkosyl extraction method. The lipid profiles of WC and OM fractions were analyzed by LC-MS/MS. The same results are displayed in Fig. 5D but are expanded here to show individual lipid names. The color of each bar reflects the base 2 logarithm of the OM vs. WC fold change for that PL in that strain.

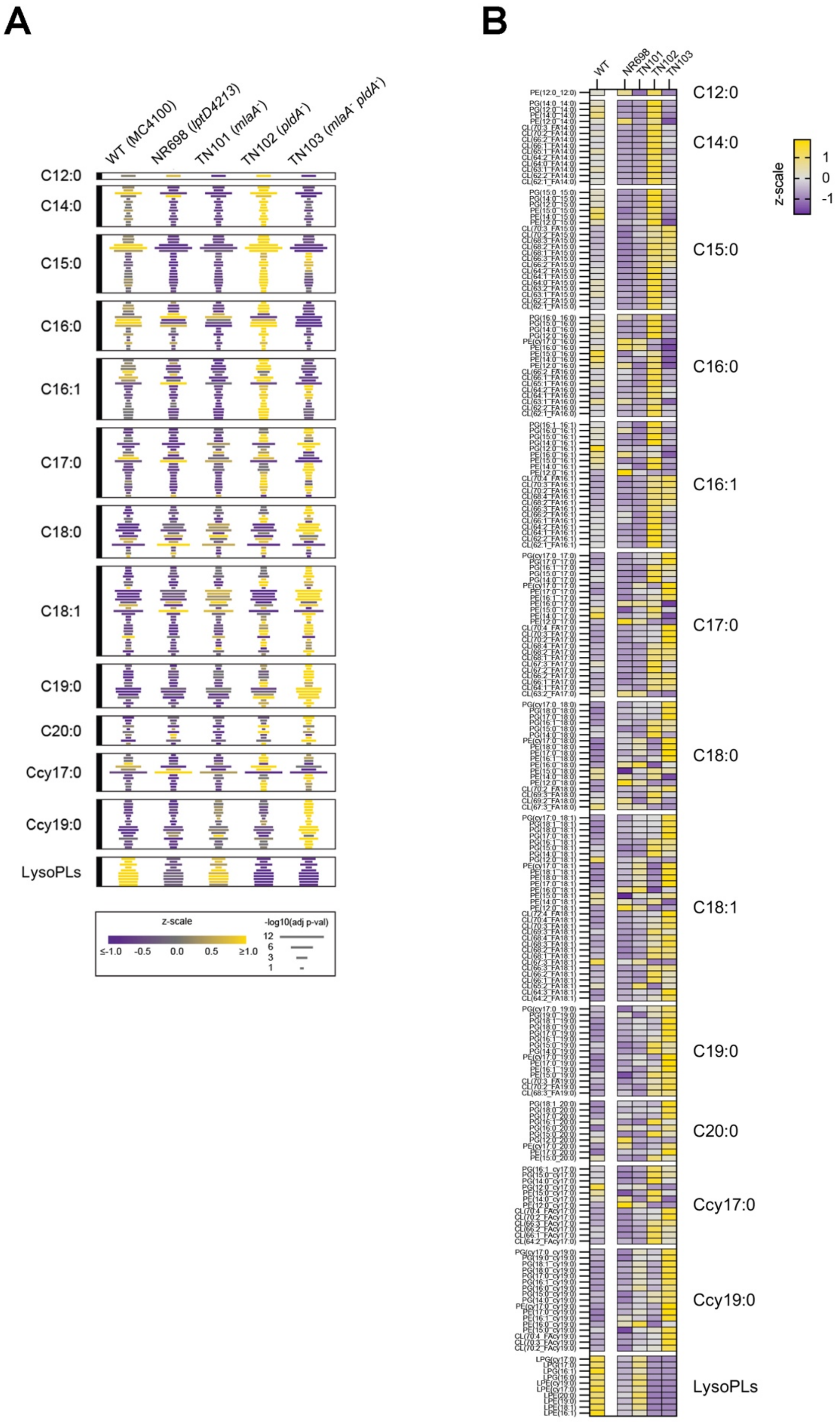
A
WT (MC4100)
NR698 (lptD4213)
TN101 (mlaA-)
TN102 (pldA-)
TN103 (mlaA- pldA-)
C12:0
C14:0
C15:0
C16:0
C16:1
C17:0
C18:0
C18:1
C19:0
C20:0
Ccy17:0
Ccy19:0
LysoPLs
z-scale
≤-1.0 -0.5 0.0 0.5 ≥1.0
-log10(adj p-val)
12
6
3
1
B
WT
NR698
TN101
TN102
TN103
C12:0
C14:0
C15:0
C16:0
C16:1
C17:0
C18:0
C18:1
C19:0
C20:0
Ccy17:0
Ccy19:0
LysoPLs
z-scale
1
0
-1

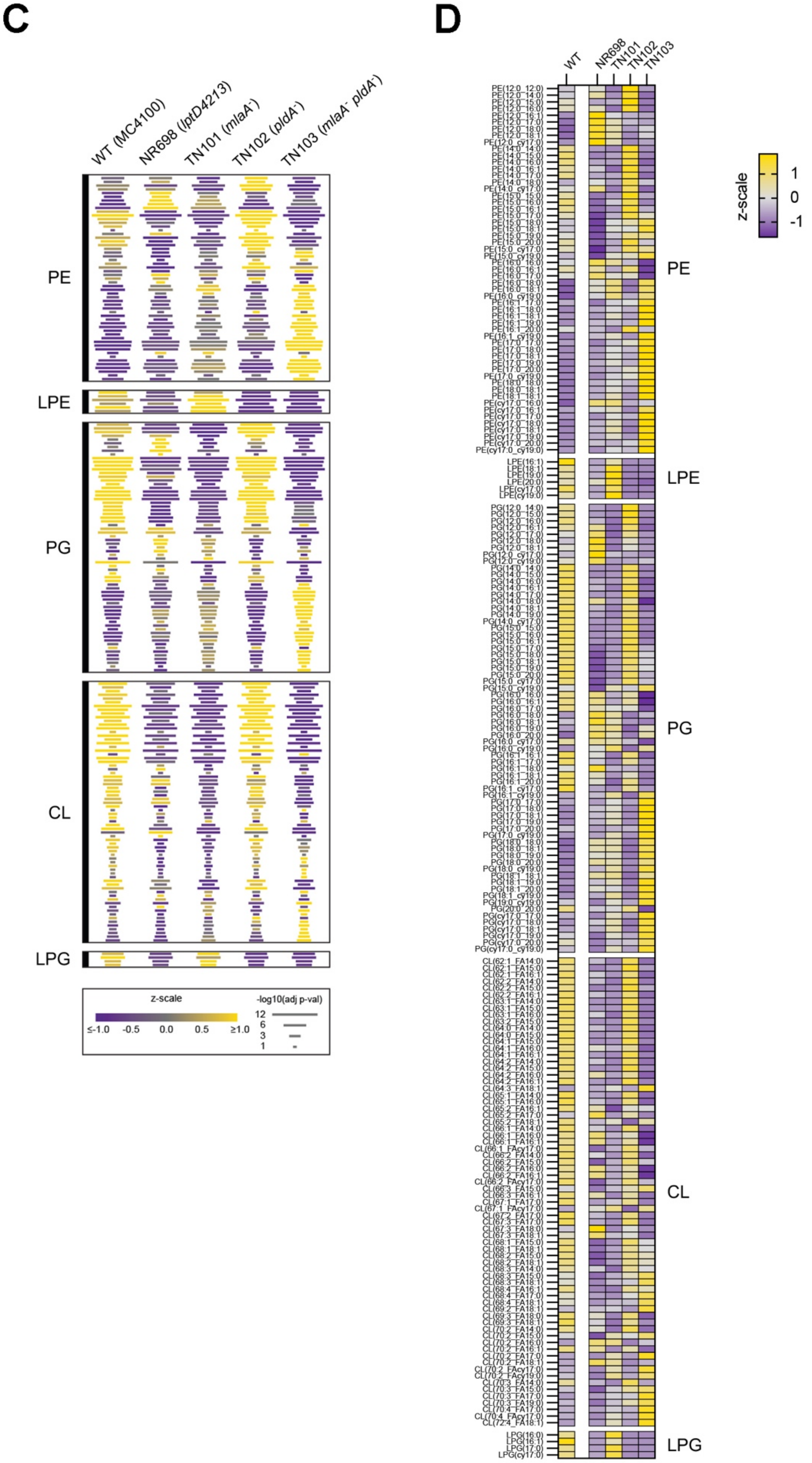
C
WT (MC4100)
NR698 (lptD4213)
TN101 (mlaA-)
TN102 (pldA-)
TN103 (mlaA- pldA-)
PE
LPE
PG
CL
LPG
z-scale
≤-1.0 -0.5 0.0 0.5 ≥1.0
-log10(adj p-val)
12
6
3
1
D
WT
NR698
TN101
TN102
TN103
z-scale
1
0
-1
PE
LPE
PG
CL
LPG

E

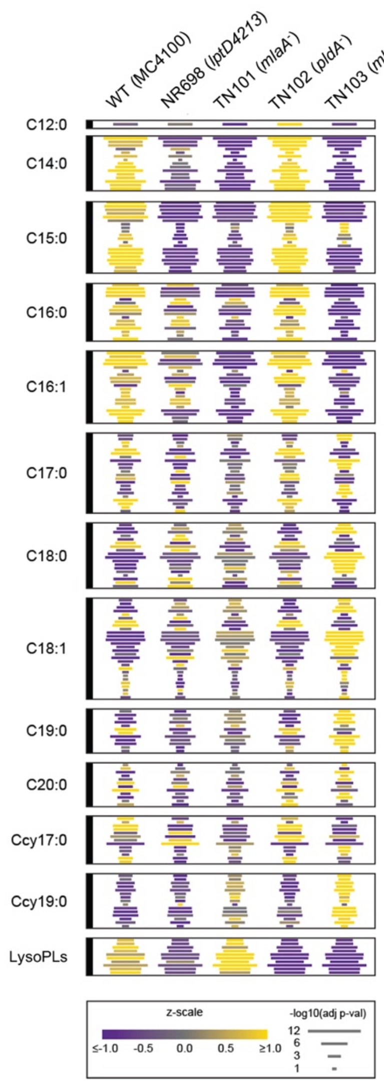
WT (MC4100)
NR698 (lptD4213)
TN101 (mlaA⁻)
TN102 (pldA⁻)
TN103 (mlaA⁻ pldA⁻)
C12:0
C14:0
C15:0
C16:0
C16:1
C17:0
C18:0
C18:1
C19:0
C20:0
Ccy17:0
Ccy19:0
LysoPLs
z-scale
≤-1.0
-0.5
0.0
0.5
≥1.0
-log10(adj p-val)
12
6
3
1

F

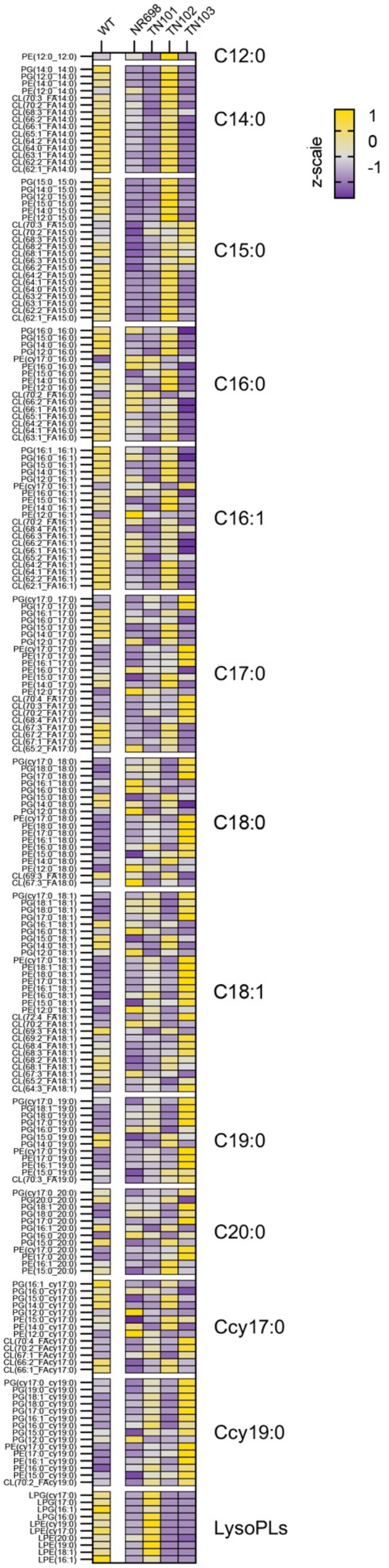
WT
NR698
TN101
TN102
TN103
C12:0
C14:0
C15:0
C16:0
C16:1
C17:0
C18:0
C18:1
C19:0
C20:0
Ccy17:0
Ccy19:0
LysoPLs
z-scale
1
0
-1

Fig. S14. Expanded PL profiles of purified OMs and WCs organized by lipid class and acyl chain length. (A) The one-way ANOVA analysis of the OM phospholipidome displayed in Figs. 6A and 6B is shown, but arranged by the acyl chain length in ascending order. (B) The analysis shown in a was expanded to show individual PLs on a heat map. (C) A comparison of the WC phospholipidome across strains by one-way ANOVA. PLs that passed a filter for multiple comparisons using the Benjamini Hochberg method set at an FDR of 10% are displayed. The color of each bar reflects the z-scaled abundance within the WC dataset and the length of each row of bars reflects the negative base 10 logarithm of the ANOVA p-value and is thus shared across all strains for a specific lipid. Within each lipid class individual PLs are displayed by acyl chain length in an ascending order. (D) The analysis shown in C was expanded to show individual PLs on a heat map. (E) The same one-way ANOVA analysis of the WC phospholipidome displayed in C and D is shown but is arranged only by the acyl chain length in ascending order. (F) The analysis shown in E was expanded to show individual lipids on a heat map.

Supplementary References:

1. Hussain, S. & Bernstein, H. D. The Bam complex catalyzes efficient insertion of bacterial outer membrane proteins into membrane vesicles of variable lipid composition. *J Biol Chem* **293**, 2959-2973 (2018).
2. Wang, X., Nyenhuis, S. B. & Bernstein, H. D. The translocation assembly module (TAM) catalyzes the assembly of bacterial outer membrane proteins in vitro. *Nat Commun* **15**, 7246 (2024).
3. Ruiz, N., Falcone, B., Kahne, D. & Silhavy, T. J. Chemical conditionality: a genetic strategy to probe organelle assembly. *Cell* **121**, 307-317 (2005).
4. Braun, M. & Silhavy, T. J. Imp/OstA is required for cell envelope biogenesis in *Escherichia coli*. *Mol Microbiol* **45**, 1289-1302 (2002).
