# Supplementary material for: Phospholipid composition strongly affects the assembly of β barrel proteins into purified bacterial outer membranes
Source: Nat Commun. 2026 Jan 21;17:1915. doi: 10.1038/s41467-026-68743-3 (PMC12923597; doi:10.1038/s41467-026-68743-3)
Supplement: Supplementary file 2 — Description of Additional Supplementary Files [file 41467_2026_68743_MOESM2_ESM.pdf]

Description of Additional Supplementary Files:

Supplementary Data 1: Oligonucleotides used in this study.

Supplementary Data 2: Filtered and normalized lipidomics data from all samples described in this study.
