# Supplementary material for: Phospholipid composition strongly affects the assembly of β barrel proteins into purified bacterial outer membranes
Source: Nat Commun. 2026 Jan 21;17:1915. doi: 10.1038/s41467-026-68743-3 (PMC12923597; doi:10.1038/s41467-026-68743-3)
Supplement: Supplementary file 7 — Source Data [file 41467_2026_68743_MOESM7_ESM.zip › Source Data.1.pdf]

Main manuscript related data

Fig. 2. BAM present in native OMs purified by sarkosyl extraction catalyzes OMP assembly.
Uncropped gels

C

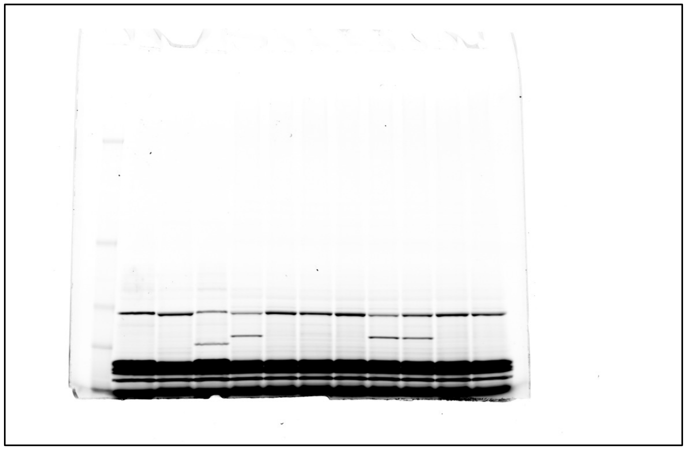

D

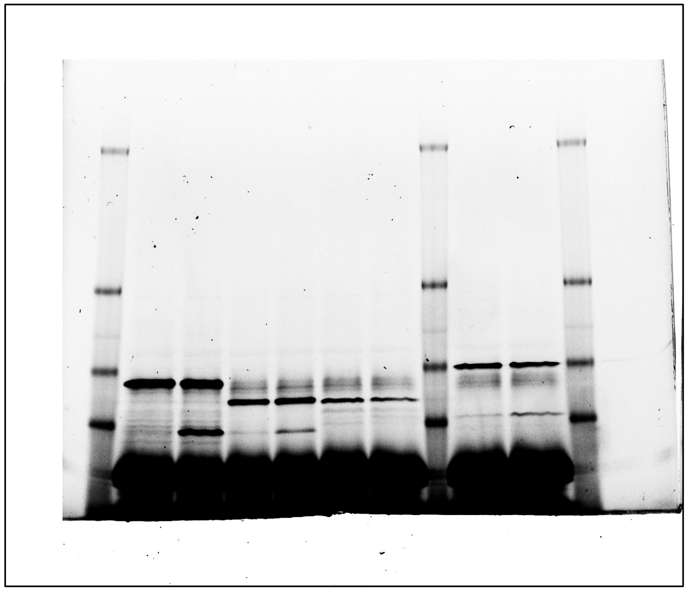

Fig. 2. BAM present in native OMs purified by sarkosyl extraction catalyzes OMP assembly.
Uncropped gel, raw data

E

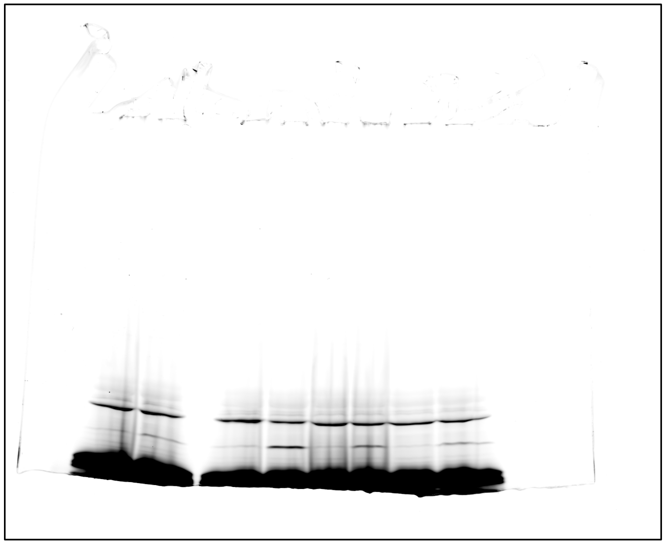

EspPΔ5' band intensity	S
β barrel band intensity	P
Total intensity	S+P
folding fraction	P/(S+P)

Sample	S	P	S+P	P/(S+P)	% folding
MC4100 (NI)	6758.74	959.77	7718.51	0.12434654	12.4346538
MC4100 (I)	7036.569	5735.669	12772.238	0.44907314	44.9073138

Fig. 3. The efficiency of EspPΔ5' folding is reduced in the presence of native OMs purified from mutant strains deficient in OM lipid homeostasis.

Uncropped gel, raw data

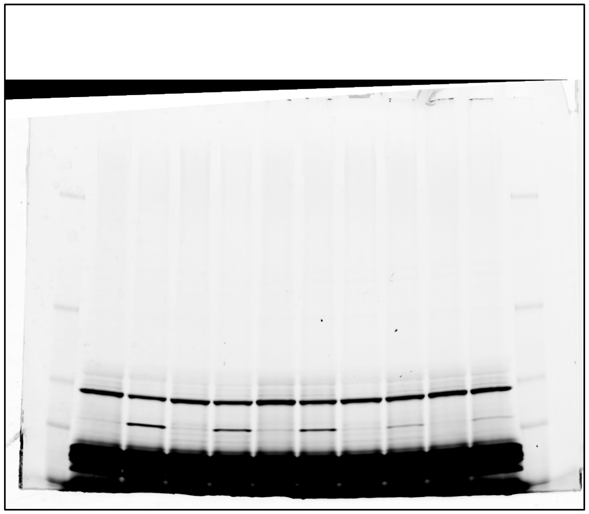

EspPΔ5' band intensity	S
β barrel band intensity	P
Total intensity	S+P
folding fraction	P/(S+P)

Sample	S	P	S+P	% folding
MC4100	5564.711	3367.205	8931.916	37.6985744
NR698	7676.347	2183.962	9860.309	22.1490219
mlaA-	7188.054	2006.891	9194.945	21.8260251
pldA-	8109.882	584.991	8694.873	6.72799936
mlaA- pldA-	9533.882	369.92	9903.802	3.73513122

Fig. 4. Kinetics of EspPΔ5' assembly into native OMs purified from MC4100 and mutant strains.
Uncropped gels

A

No OM - control

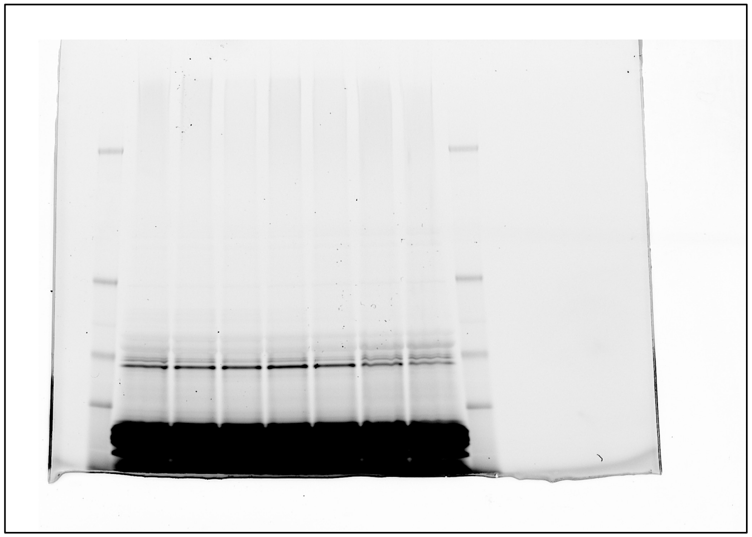

MC4100

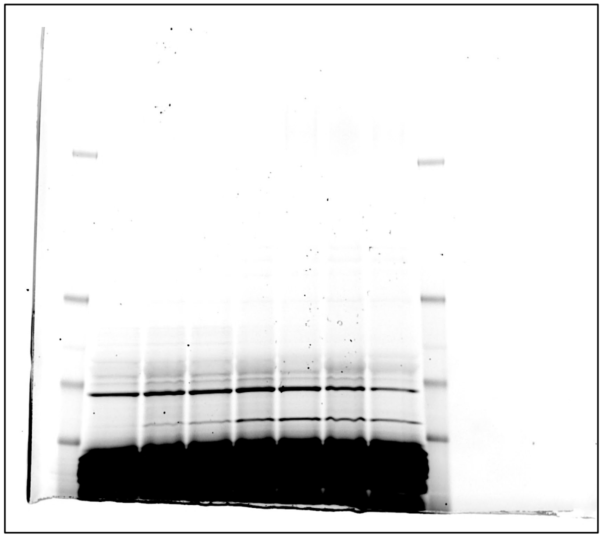

Fig. 4. Kinetics of EspPΔ5' assembly into native OMs purified from MC4100 and mutant strains.

B

NR698 (*lptD4213*)

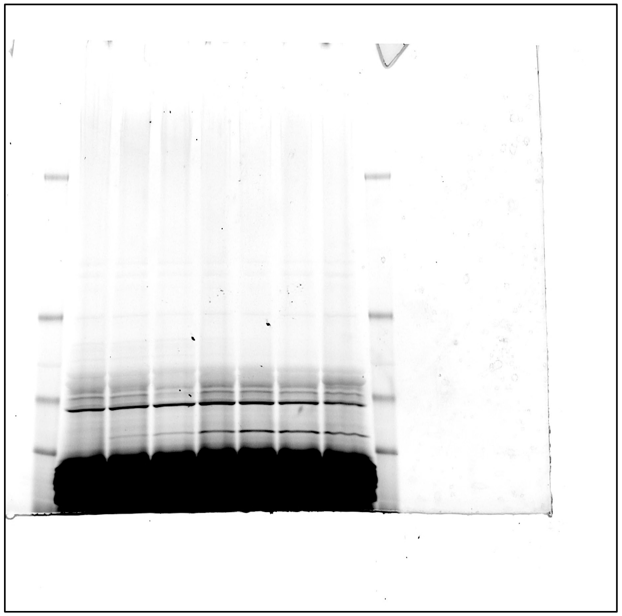

TN102 (*pldA*[-])

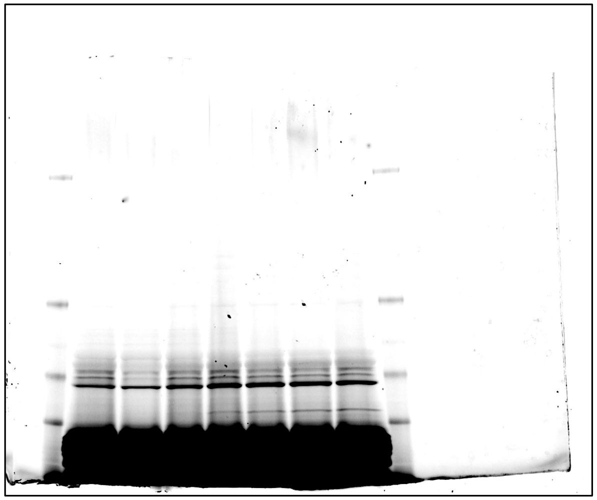

Fig. 4. Kinetics of EspPΔ5' assembly into native OMs purified from MC4100 and mutant strains.

B

TN101 (*mlaA*$^{-}$)

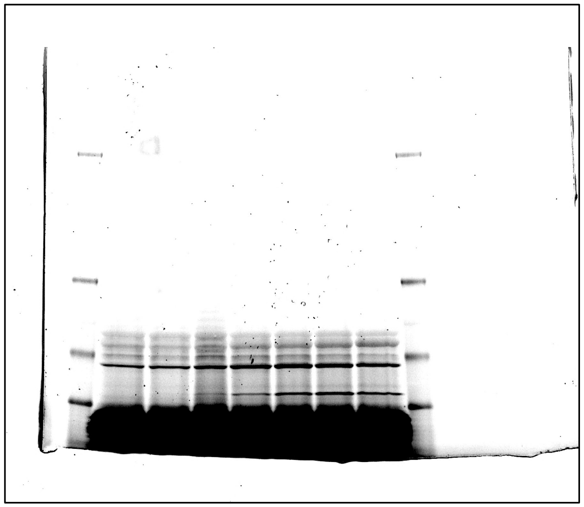

TN103 (*mlaA*$^{-}$ *pldA*$^{-}$)

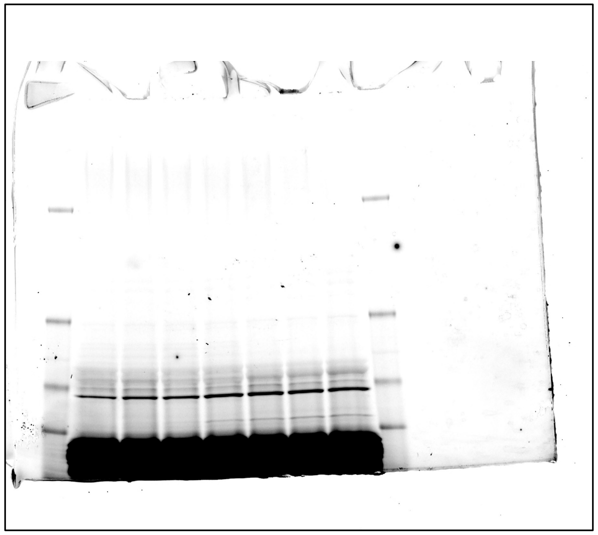

Fig. 4. Kinetics of EspPΔ5' assembly into native OMs purified from MC4100 and mutant strains.

Raw data used in 4A and 4B

EspPΔ5' band intensity	S
β barrel band intensity	P
Total intensity	S+P
folding fraction	P/(S+P)

MC4100

Time point	S	P	S+P	P/(S+P)	Folding %
0.25	8800.711	0	8800.711	0	0
0.5	6107.054	462.506	6569.56	0.07040137	7.04013663
1	6705.054	766.648	7471.702	0.10260688	10.2606876
2.5	7612.761	1915.255	9528.016	0.20101299	20.1012992
5	8281.518	2528.255	10809.773	0.23388604	23.388604
15	4033.276	1977.669	6010.945	0.32901133	32.9011329
30	4265.397	2837.69	7103.087	0.39950095	39.9500949

NR698

Time point	S	P	S+P	P/(S+P)	Folding %
0.25	4925.64	0	4925.64	0	0
0.5	5730.004	292.263	6022.267	0.0485304	4.85303956
1	6677.468	604.113	7281.581	0.08296454	8.29645375
2.5	5572.933	1162.77	6735.703	0.17262786	17.2627861
5	4754.104	1762.719	6516.823	0.27048748	27.0487475
15	4929.518	2564.669	7494.187	0.34222111	34.2221111
30	9720.945	5123.782	14844.727	0.34515839	34.5158385

TN101 - mlaA

Time point	S	P	S+P	P/(S+P)	Folding %
0.25	10362.681	0	10362.681	0	0
0.5	3236.861	0	3236.861	0	0
1	7804.125	377.87	8181.995	0.04618311	4.6183113
2.5	5762.711	794.184	6556.895	0.12112196	12.1121964
5	7546.347	1887.255	9433.602	0.20005667	20.005667
15	6675.933	2845.083	9521.016	0.29882137	29.8821365
30	9070.296	4227.811	13298.107	0.31792578	31.7925777

TN102 - pldA

Time point	S	P	S+P	P/(S+P)	Folding %
0.25	3601.004	0	3601.004	0	0
0.5	3734.397	0	3734.397	0	0
1	8402.903	43.364	8446.267	0.0051341	0.51341024
2.5	8749.196	295.678	9044.874	0.03269012	3.26901182
5	8871.368	463.577	9334.945	0.04966039	4.9660389
15	9269.489	693.941	9963.43	0.06964881	6.96488057
30	8598.368	819.062	9417.43	0.08697299	8.69729852

TN103 - mlaA pldA

Time point	S	P	S+P	P/(S+P)	Folding %
0.25	7870.933	0	7870.933	0	0
0.5	4345.154	0	4345.154	0	0
1	4960.74	0	4960.74	0	0
2.5	7868.104	280.263	8148.367	0.03439499	3.43949898
5	7724.347	405.335	8129.682	0.04985865	4.98586538
15	9172.246	721.355	9893.601	0.07291127	7.29112686
30	10234.711	959.012	11193.723	0.08567409	8.56740872

Fig. 4. Kinetics of EspPΔ5' assembly into native OMs purified from MC4100 and mutant strains.

Data used to generate the 4C plot, calculate the rate constant, $t_{1/2}$ and p values.

time (min)	MC4100 BAM-OM				NR698 BAM-OM				mlaA- BAM-OM				pldA- BAM-OM				mlaA- pldA- BAM-OM			
	OM Prep1		OM Prep 2		OM Prep1		OM Prep 2		OM Prep1		OM Prep 2		OM Prep1		OM Prep 2		OM Prep1		OM Prep 2	
	rx1	rx2	rx1	rx2	rx1	rx2	rx1	rx2	rx1	rx2	rx1	rx2	rx1	rx2	rx1	rx2	rx1	rx2	rx1	rx2
0.25	0	0	0	0	0	0	0	0	0	0	0	0	0	0	0	0	0	0	0	0
0.5	7.04	6.67	7.24	8.92	2.61	1.67	5.47	4.85	3.19	2.60	0.00	1.79	0.00	0.00	0.00	0.00	0.00	0.00	0.00	0.00
1	10.26	11.78	10.90	11.29	6.32	7.06	9.37	8.30	5.71	5.20	4.62	5.97	2.16	0.51	0.94	0.00	0.00	0.00	1.55	0.00
2.5	20.10	22.09	21.16	20.85	13.89	13.60	19.72	17.26	15.52	11.21	12.11	15.09	3.26	3.27	2.46	2.42	3.21	3.03	3.54	3.44
5	23.39	29.84	22.09	27.71	19.20	16.86	30.33	27.05	18.42	17.75	20.01	20.34	4.77	4.97	3.84	3.50	4.96	4.66	5.59	4.99
15	32.90	38.11	31.53	34.08	24.21	24.06	33.75	34.22	27.58	26.94	29.88	27.34	6.22	6.96	5.90	6.29	6.21	6.40	7.39	7.29
30	39.95	39.49	36.05	39.06	24.97	25.28	33.59	34.52	31.06	29.58	31.79	30.04	8.17	8.70	6.57	6.85	6.62	6.91	8.76	8.57

Supplementary Information related data

Fig. S1. Characterization of BAM in OMs purified by sarkosyl extraction.
Uncropped blots

A

BamA

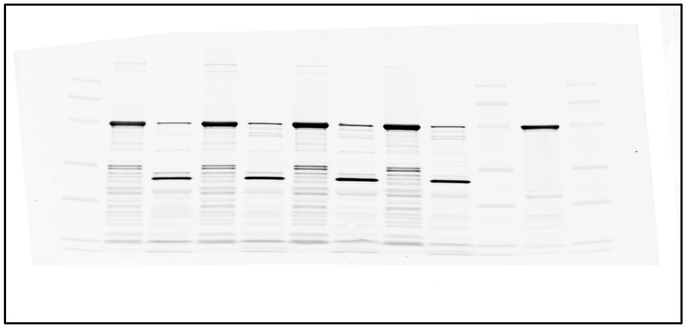

B

BamB

BamD

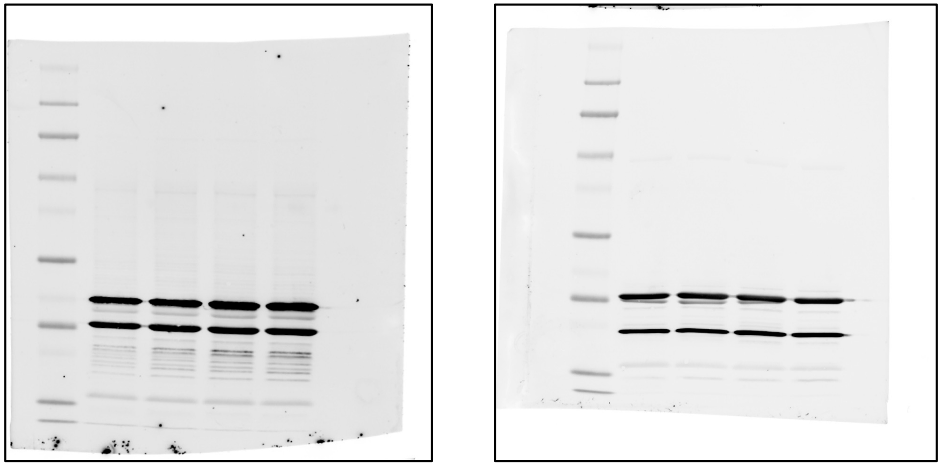

Fig. S1. Characterization of BAM in OMs purified by sarkosyl extraction.

Uncropped gel and raw data

D

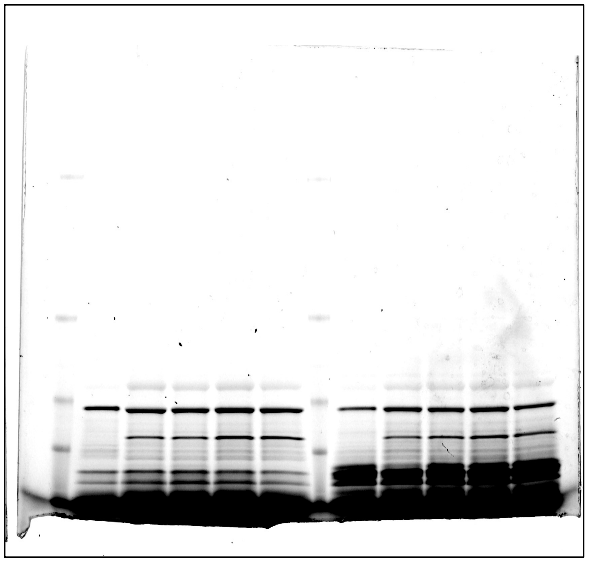

EspPΔ5' band intensity	S
β barrel band intensity	P
Total intensity	S+P
folding fraction	P/(S+P)

Sample	S	P	S+P	P/(S+P)	% folding
Control (no OM)	8122.054		8122.054	0	0
LB	5572.054	1250.648	6822.702	0.18330685	18.3306848
LB(C+)	5396.225	1212.355	6608.58	0.18345166	18.3451664
LB+MC	7609.589	2435.426	10045.015	0.24245121	24.2451206
LB+MC (C+)	9376.539	3404.255	12780.794	0.26635708	26.6357082

Fig. S2. Characterization of BAM in OMs purified by the sucrose gradient method.
Uncropped blots

A

BamA

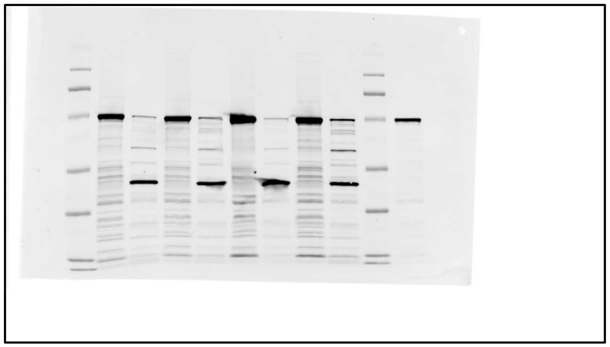

BamA

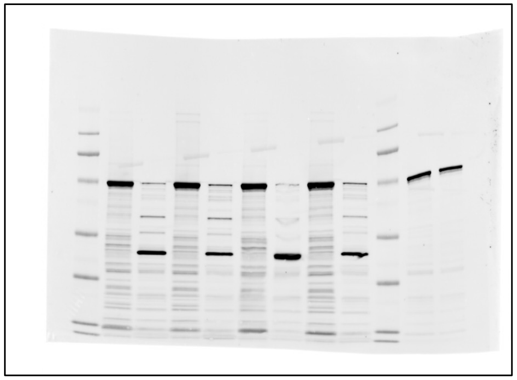

70% sucrose gradient (left) and a 73% sucrose gradient (right)

Fig. S2. Characterization of BAM in OMs purified by the sucrose gradient method.
Uncropped blots

B

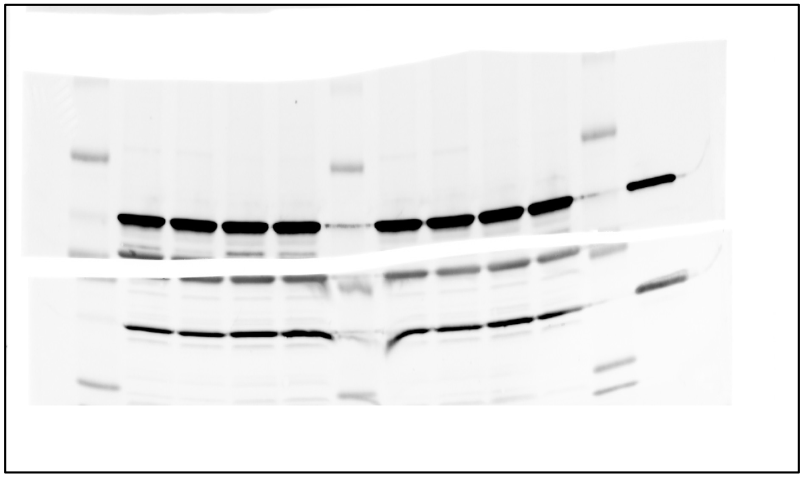

BamB

BamD

70% sucrose gradient (left) and a 73% sucrose gradient (right)

Fig. S3. Quantitation of BAM in native OMs.
Uncropped blots and raw data

A

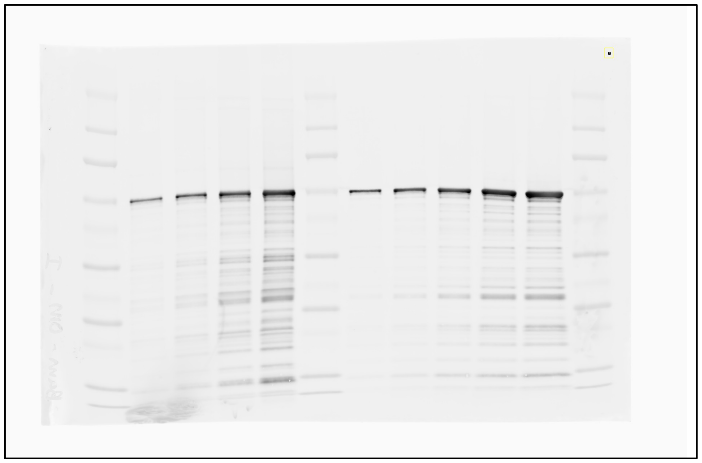

BAM induced

Induced				
BAM-DDM	Signal intensity		I sample	Signal intensity
60	12456.225		x	10008.761
40	10952.933		0.5x	7055.104
20	7128.569		0.25x	4670.033
10	5095.619			
5	3677.598			

Fig. S3. Quantitation of BAM in native OMs.

Uncropped blots and raw data

B

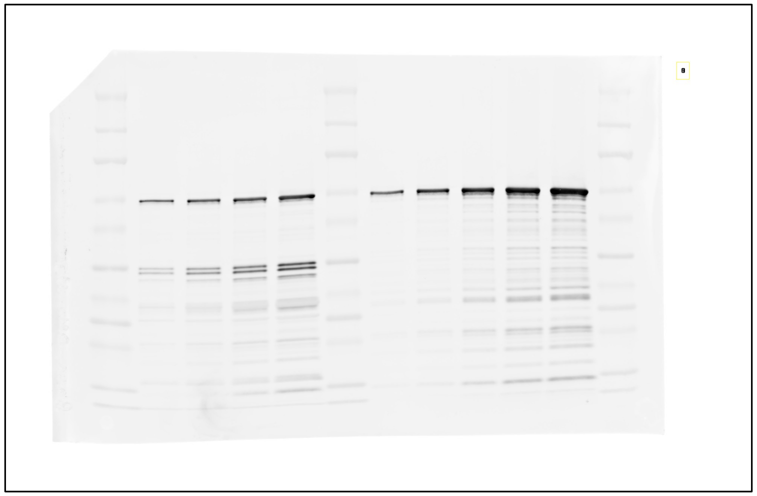

BAM non-induced

Non-induced				
BAM-DDM	Signal intensity		NI sample	Signal intensity
60	13340.296		x	6424.447
40	11160.589		0.5x	4402.426
20	8180.64		0.25x	3456.891
10	5337.326			
5	3065.598			

Fig. S4. The assembly of *de novo* synthesized EspPΔ5' into native OMs purified by sarkosyl extraction is highly reproducible.

Uncropped gels

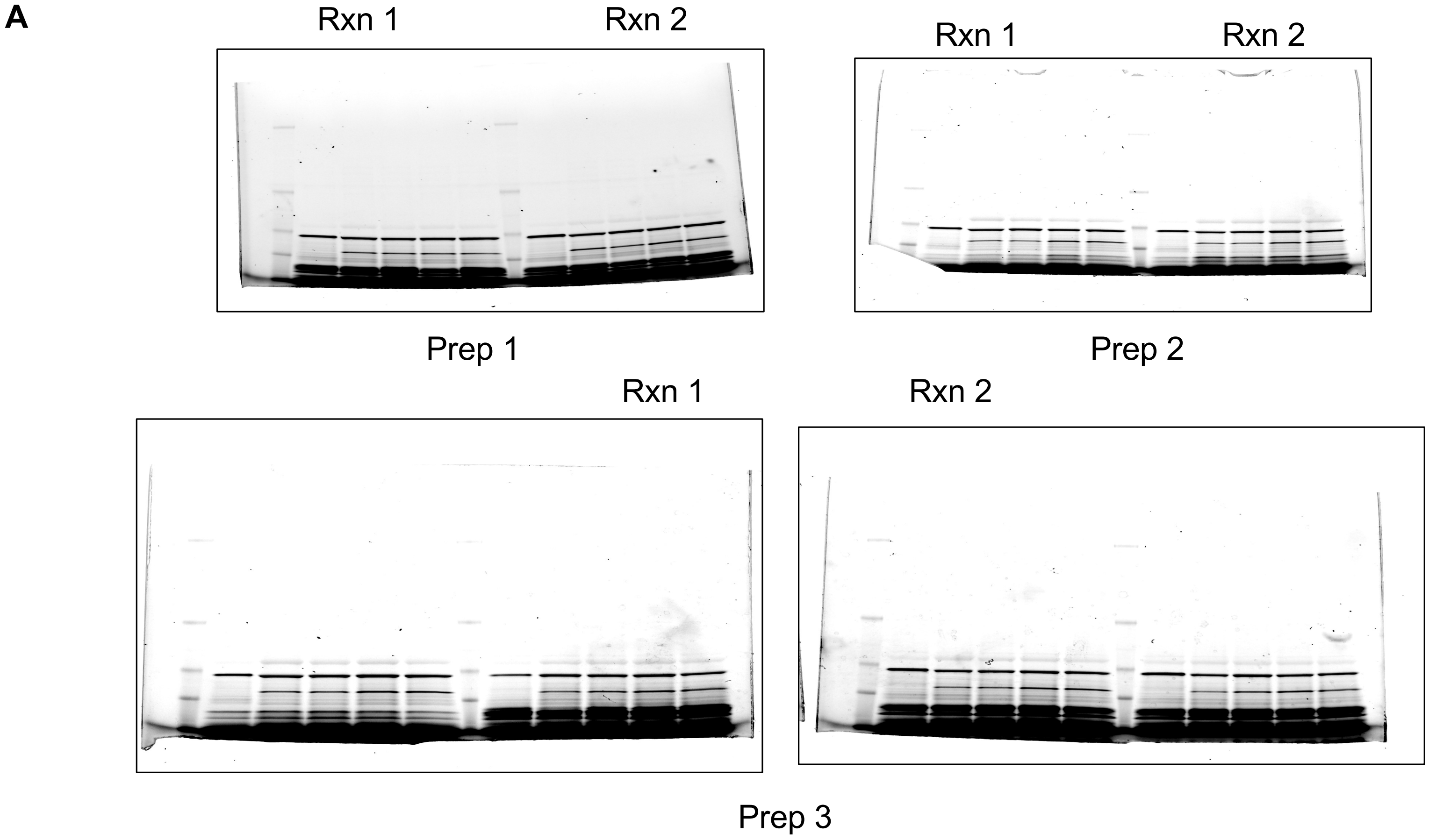

Fig. S4. The assembly of *de novo* synthesized EspPΔ5' into native OMs purified by sarkosyl extraction is highly reproducible.

Uncropped gels

B

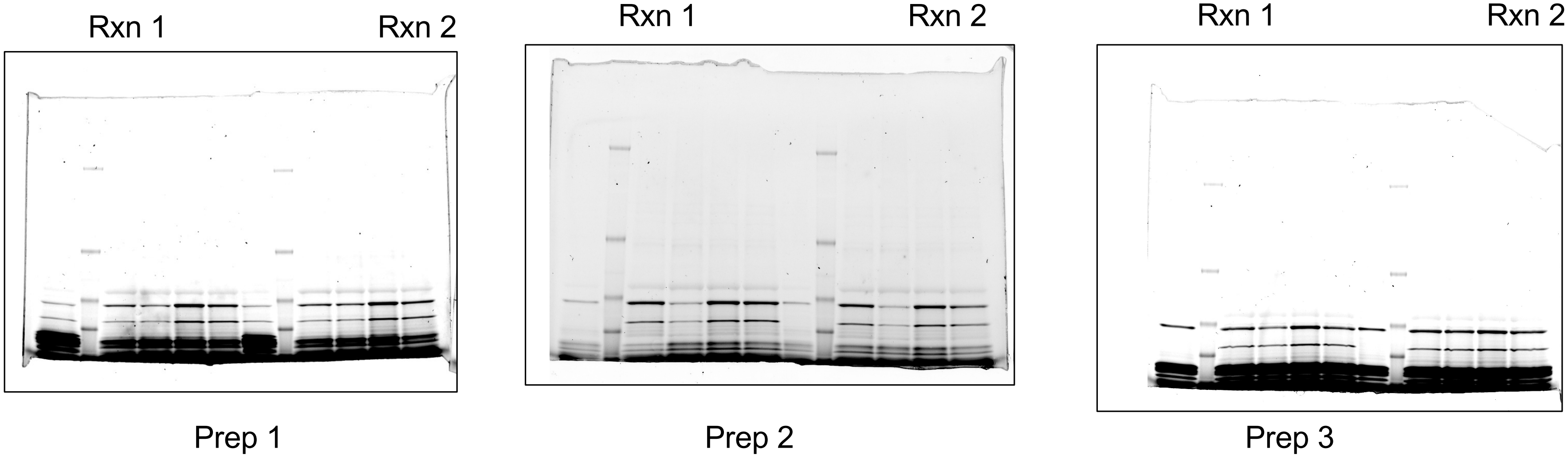

Fig. S4. The assembly of *de novo* synthesized EspPΔ5' into native OMs purified by sarkosyl extraction is highly reproducible.

Uncropped gels

C

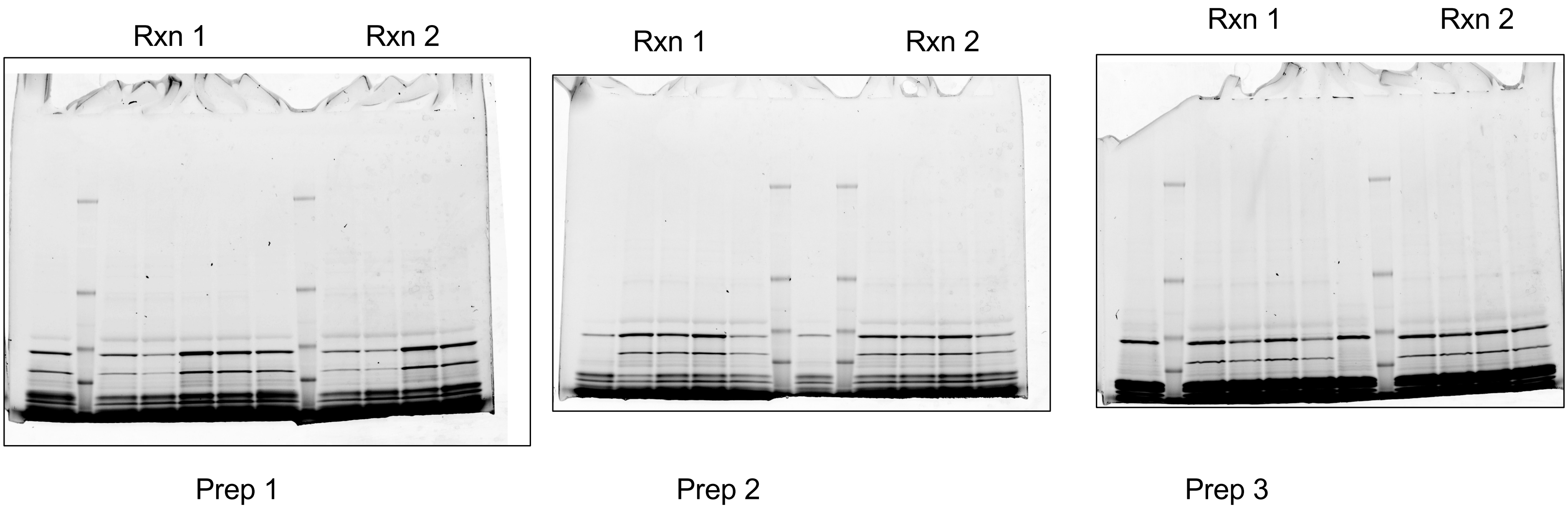

Fig. S5. The effect of varying the concentration of SurA on the assembly of *de novo* synthesized EspPΔ5'.

Uncropped gels and raw data

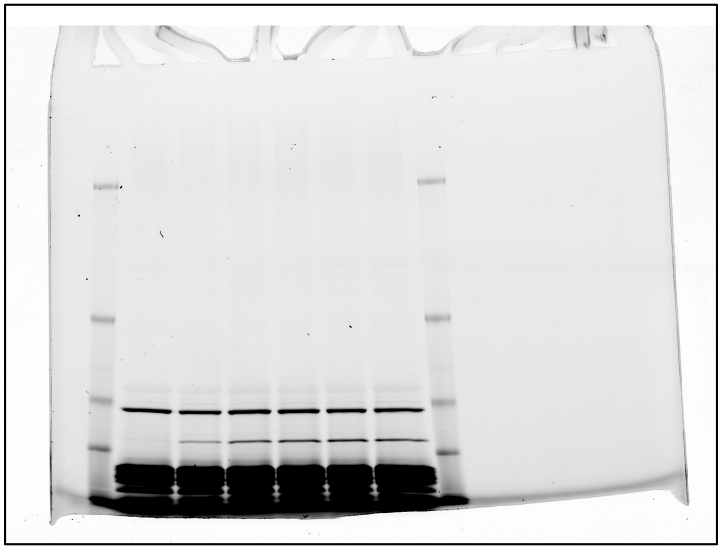

EspPΔ5' band intensity	S
β barrel band intensity	P
Total intensity	S+P
folding fraction	P/(S+P)

SurA concentration	S	P	P/(S+P)	% folding
0	10469.468	0	0	0
1	7577.811	1022.648	0.11890621	11.8906212
2	9775.054	2235.426	0.18612295	18.6122953
4	9252.054	2665.962	0.22369176	22.3691762
8	7481.054	2870.548	0.27730471	27.7304711
10	9433.054	3457.255	0.26820575	26.8205751

Fig. S6. Characterization of BAM in native OMs purified by sarkosyl extraction from MC4100, NR698, TN101, TN102 and TN103.
Uncropped blots

BamA

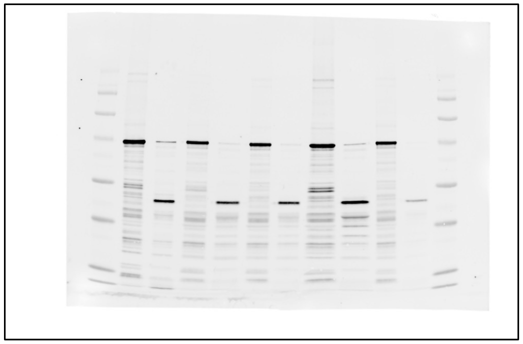

BamA

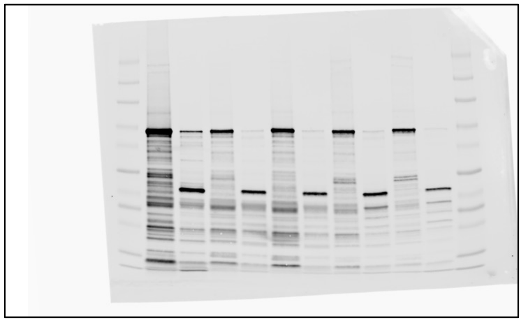

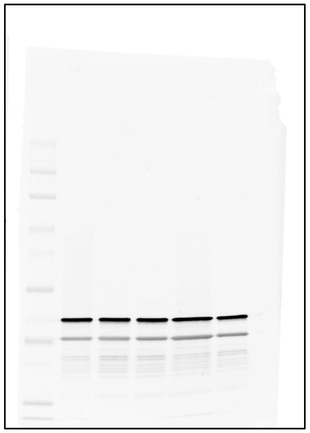

BamB

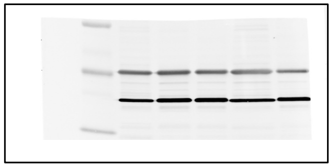

BamD

Fig. S7. Residual peptidoglycan on native OMs does not affect the assembly of *de novo* synthesized EspPΔ5'.

Uncropped gels and raw data

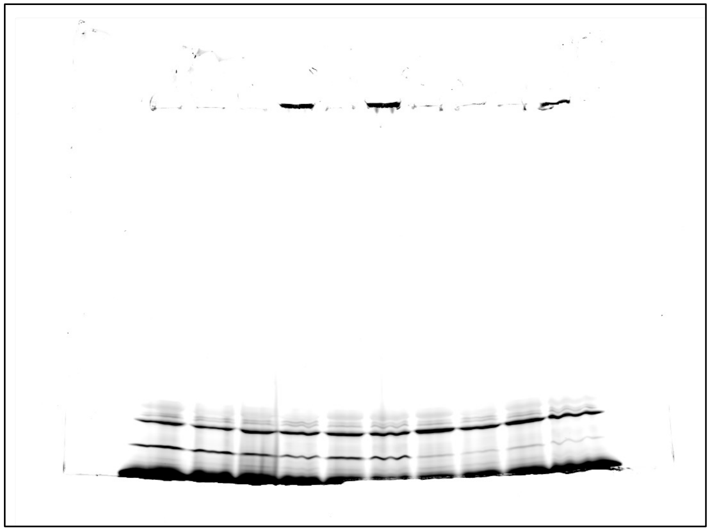

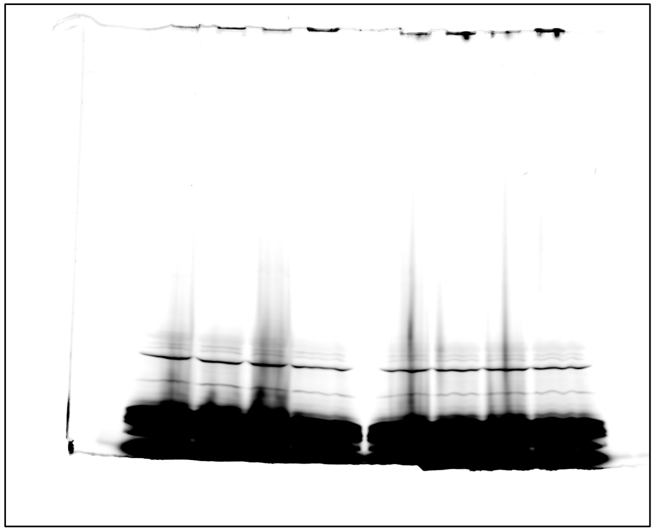

EspPΔ5' band intensity	S
β barrel band intensity	P
Total intensity	S+P
folding fraction	P/(S+P)

Sample	S	P	S+P	P/(S+P)	% folding
MC4100 control	9417.64	6461.205	15878.845	0.406906485	40.6906485
MC4100 (lyso+)	7705.154	5408.962	13114.116	0.41245342	41.245342
NR698 control	3913.426	1326.77	5240.196	0.253190911	25.3190911
NR698 (lyso+)	3720.426	1231.527	4951.953	0.248695212	24.8695212
TN101 control	4229.962	1634.698	5864.66	0.278737045	27.8737045
TN101 (lyso+)	4496.205	1618.82	6115.025	0.264728272	26.4728272
TN102 control	7172.79	320.92	7493.71	0.042825249	4.28252494
TN102 (lyso+)	4675.497	221.849	4897.346	0.045299842	4.5299842
TN103 control	6938.74	361.971	7300.711	0.04958024	4.95802395
TN103 (lyso+)	13065.368	500.042	13565.41	0.036861547	3.68615471

Sample	S	P	S+P	P/(S+P)	% folding
NI control	7320.64	938.891	8259.531	0.11367365	11.3673646
NI (lyso+)	6067.912	986.062	7053.974	0.13978815	13.9788153

Fig. S8. Assembly assay results repeats used for Fig. 4C.
Uncropped gels

A

MC4100 OM prep 1

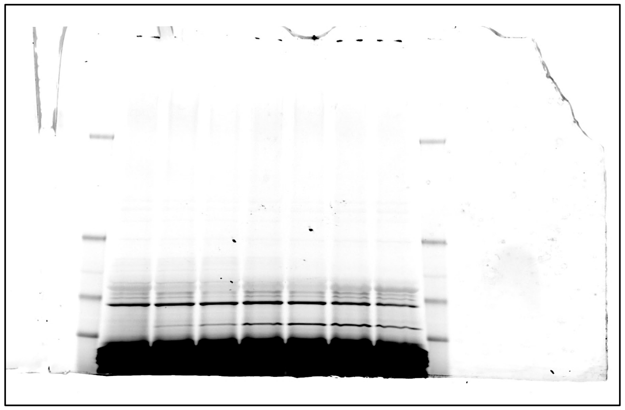

Rxn 2

MC4100 OM prep 2

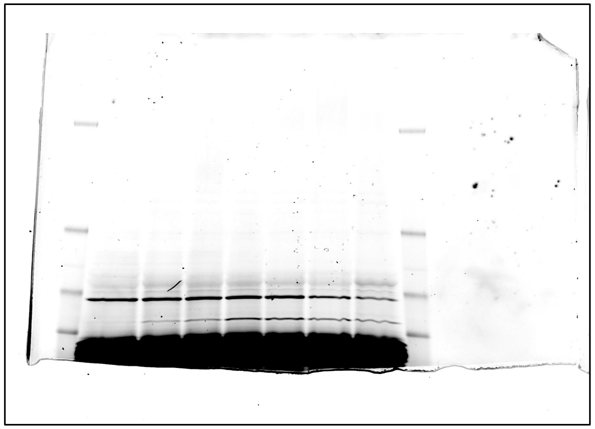

Rxn 1

MC4100 OM prep 2

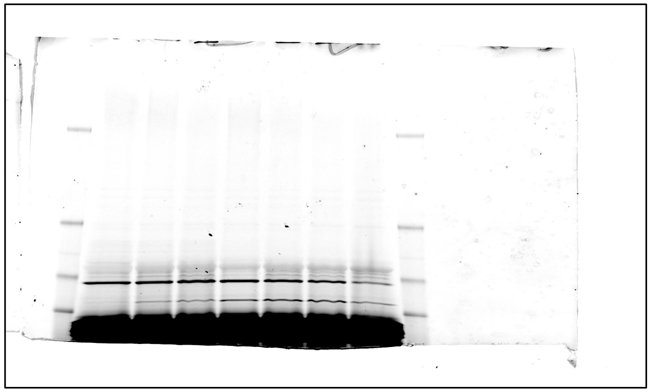

Rxn 2

Fig. S8. Assembly assay results repeats used for Fig. 4C.
Uncropped gels

B

NR698 (*lptD4213*) OM prep 1

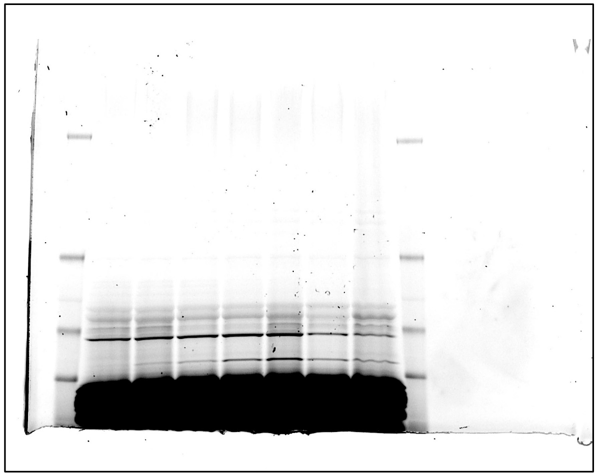

Rxn 2

NR698 OM prep 2

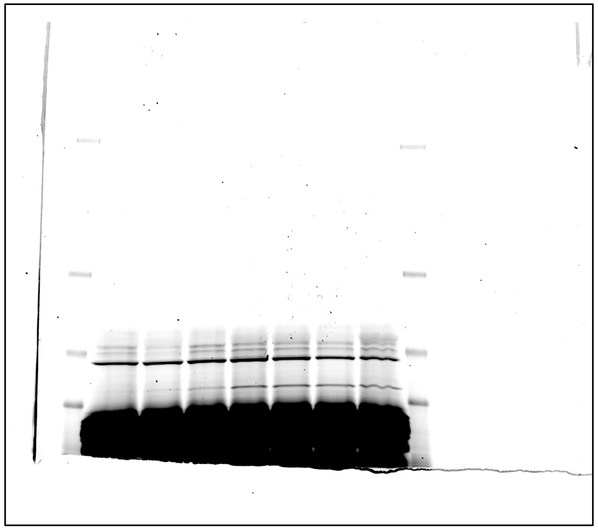

Rxn 1

NR698 OM prep 2

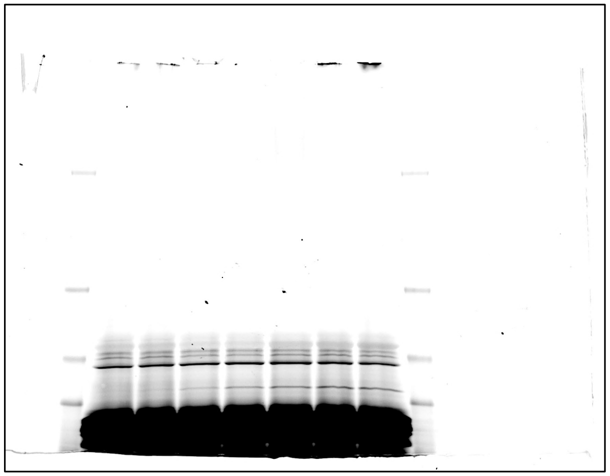

Rxn 2

Fig. S8. Assembly assay results repeats used for Fig. 4C.
Uncropped gels

C

TN101 (*mlaA*$^-$) OM prep 1

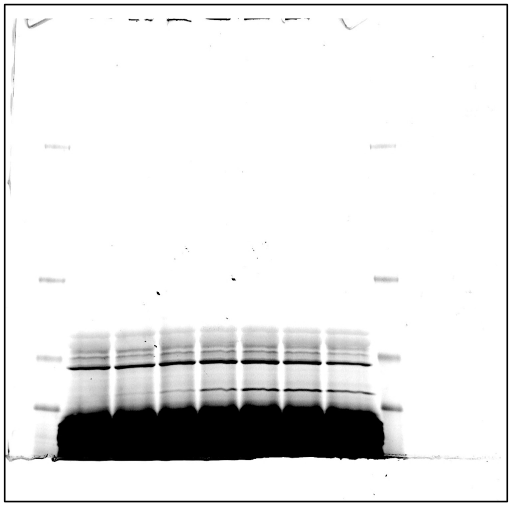

Rxn 2

TN101 OM prep 2

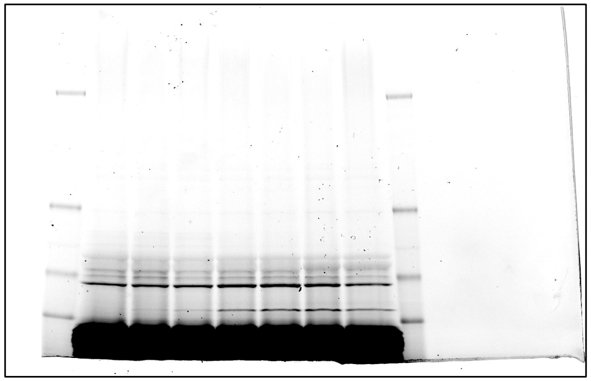

Rxn 1

TN101 OM prep 2

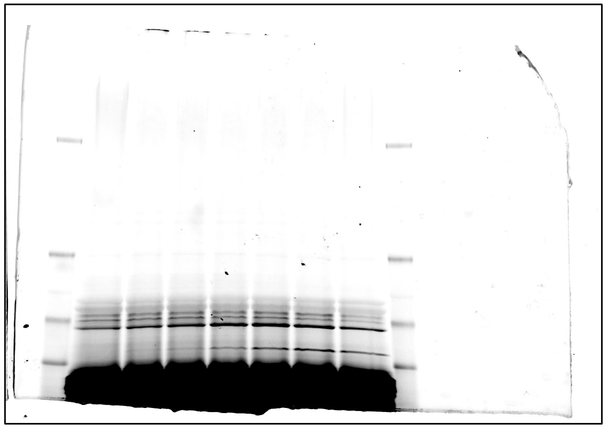

Rxn 2

Fig. S8. Assembly assay results repeats used for Fig. 4C.
Uncropped gels

D

TN102 (*pldA*$^-$) OM prep 1

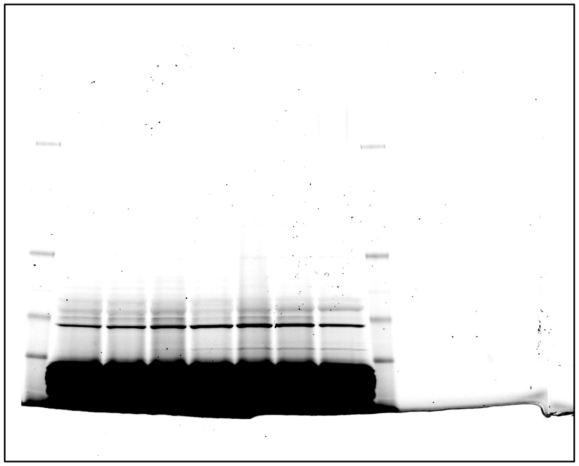

Rxn 2

TN102 OM prep 2

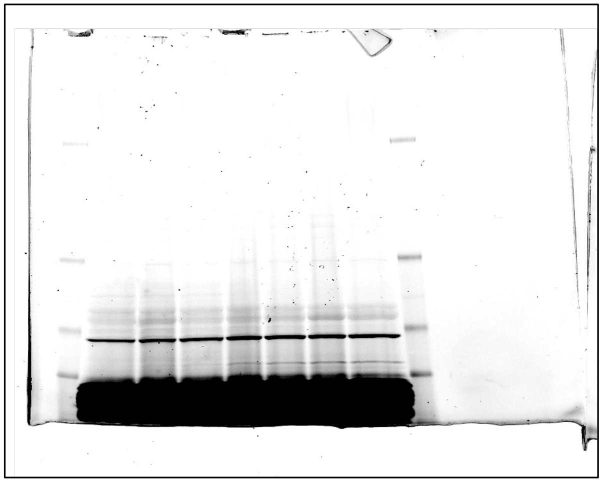

Rxn 1

TN102 OM prep 2

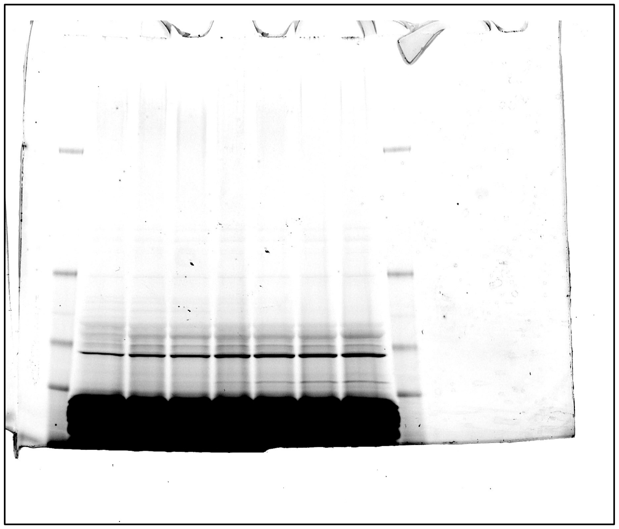

Rxn 2

Fig. S8. Assembly assay results repeats used for Fig. 4C.

Uncropped gels

E

TN103 ($mlaA^-$ $pldA^-$) OM prep 1

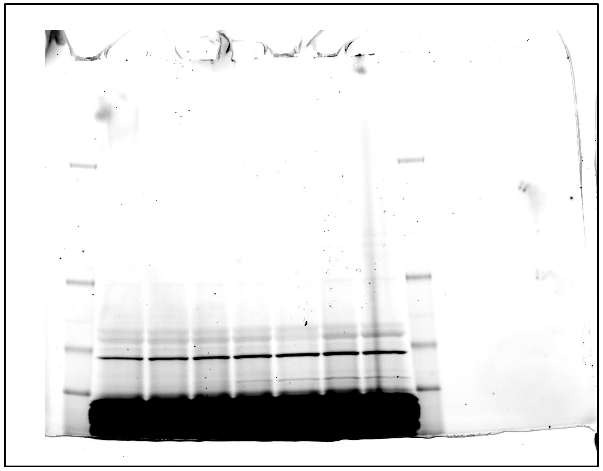

Rxn 2

TN103 OM prep 2

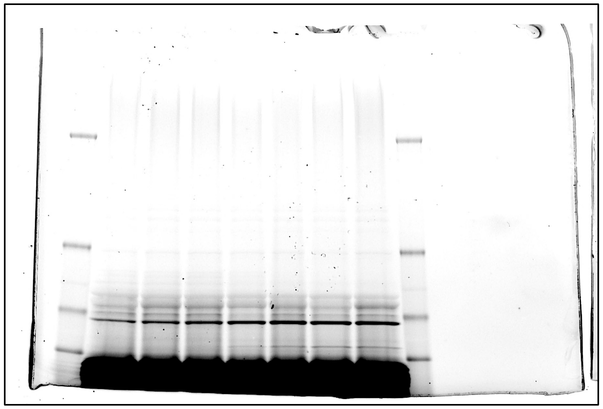

Rxn 1

TN103 OM prep 2

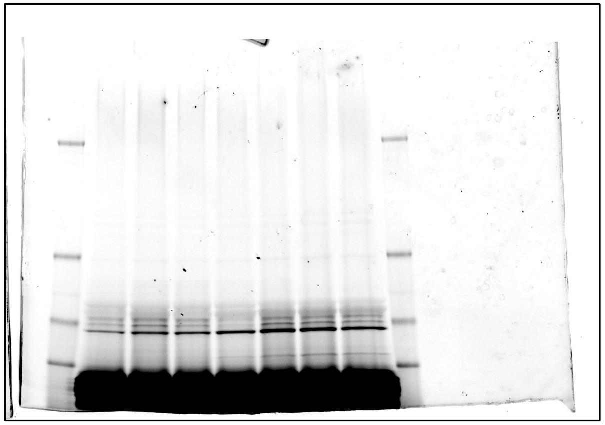

Rxn 2

Fig. S8. Assembly assay results repeats used for Fig. 4C.

Raw data

EspPΔ5' band intensity	S
β barrel band intensity	P
Total intensity	S+P
folding fraction	P/(S+P)

MC4100	prep1	rxn2			
Sample	S	P	S+P	P/(S+P)	% folding
0.25	8781.711	0	8781.711	0	0
0.5	6166.054	440.92	6606.974	0.06673554	6.67355434
1	6393.225	853.355	7246.58	0.11775969	11.7759688
2.5	7777.761	2204.841	9982.602	0.22086837	22.0868367
5	6287.104	2674.255	8961.359	0.2984207	29.8420697
15	5093.468	3136.447	8229.915	0.38110321	38.1103207
30	5124.246	3344.468	8468.714	0.39492041	39.4920409

MC4100	prep2	rxn1			
Sample	S	P	S+P	P/(S+P)	% folding
0.25	8892.175	0	8892.175	0	0
0.5	7470.347	582.991	8053.338	0.07239122	7.23912246
1	7572.054	926.477	8498.531	0.10901613	10.9016135
2.5	7656.104	2054.548	9710.652	0.21157673	21.157673
5	8021.347	2273.719	10295.066	0.22085521	22.0855214
15	8258.64	3803.619	12062.259	0.31533223	31.5332228
30	2335.983	1317.012	3652.995	0.36052937	36.0529374

MC4100	prep2	rxn2			
Sample	S	P	S+P	P/(S+P)	% folding
0.25	7775.811	0	7775.811	0	0
0.5	7129.347	698.234	7827.581	0.08920176	8.92017598
1	8230.589	1047.184	9277.773	0.11287019	11.287019
2.5	7700.69	2029.134	9729.824	0.20854786	20.8547863
5	6304.518	2417.134	8721.652	0.27714176	27.7141762
15	4734.104	2447.79	7181.894	0.34082792	34.0827921
30	1826.447	1170.891	2997.338	0.39064363	39.0643631

NR698	prep1	rxn2			
Sample	S	P	S+P	P/(S+P)	% folding
0.25	7846.539	0	7846.539	0	0
0.5	4618.933	267.435	4886.368	0.05473083	5.47308348
1	5151.69	532.577	5684.267	0.09369317	9.3693171
2.5	6393.569	1570.426	7963.995	0.19719073	19.7190732
5	5726.861	2493.548	8220.409	0.30333625	30.3336245
15	7926.64	4037.912	11964.552	0.33748961	33.7489611
30	9597.874	4854.803	14452.677	0.3359103	33.5910295

NR698	prep2	rxn1			
Sample	S	P	S+P	P/(S+P)	% folding
0.25	8166.125	0	8166.125	0	0
0.5	5831.347	156.192	5987.539	0.02608618	2.60861766
1	6654.589	448.749	7103.338	0.06317438	6.31743836
2.5	8264.66	1333.305	9597.965	0.13891538	13.8915385
5	5957.175	1415.134	7372.309	0.19195262	19.1952616
15	4160.69	1328.719	5489.409	0.24205138	24.2051376
30	3666.347	1220.134	4886.481	0.24969584	24.9695845

NR698	prep2	rxn2			
Sample	S	P	S+P	P/(S+P)	% folding
0.25	5590.66	0	5590.66	0	0
0.5	5483.125	92.95	5576.075	0.01666943	1.66694315
1	6174.246	469.335	6643.581	0.07064488	7.06448826
2.5	6800.054	1070.062	7870.116	0.13596521	13.5965213
5	7495.075	1519.891	9014.966	0.16859642	16.8596421
15	5978.125	1894.548	7872.673	0.24064863	24.0648634
30	5276.368	1785.205	7061.573	0.25280557	25.2805572

TN101-mlaA	prep1	rxn2			
Sample	S	P	S+P	P/(S+P)	% folding
0.25	8174.439	0	8174.439	0	0
0.5	7818.075	142.607	7960.682	0.01791392	1.79139174
1	4381.933	278.263	4660.196	0.05971058	5.97105787
2.5	5792.933	1029.184	6822.117	0.15085992	15.0859916
5	6551.882	1673.426	8225.308	0.20344843	20.344843
15	4694.347	1766.719	6461.066	0.27344079	27.3440791
30	9838.731	4224.589	14063.32	0.3003977	30.0397701

TN101-mlaA	prep2	rxn1			
Sample	S	P	S+P	P/(S+P)	% folding
0.25	10630.024	0	10630.024	0	0
0.5	5068.882	167.021	5235.903	0.03189918	3.18991777
1	5610.761	339.506	5950.267	0.05705727	5.70572715
2.5	8237.589	1513.598	9751.187	0.15522192	15.5221923
5	8564.782	1934.255	10499.037	0.18423166	18.4231659
15	6504.468	2477.205	8981.673	0.27580663	27.5806634
30	9546.246	4300.397	13846.643	0.31057326	31.0573256

TN101-mlaA	prep2	rxn2			
Sample	S	P	S+P	P/(S+P)	% folding
0.25	8040.137	0	8040.137	0	0
0.5	4262.196	113.778	4375.974	0.02600061	2.60006115
1	5459.196	299.263	5758.459	0.05196929	5.19692855
2.5	7938.125	1001.891	8940.016	0.11206814	11.2068144
5	8692.903	1876.134	10569.037	0.17751229	17.7512294
15	8155.196	3007.326	11162.522	0.26941277	26.9412772
30	10226.974	4296.761	14523.735	0.29584408	29.5844079

Fig. S8. Assembly assay results repeats used for Fig. 4C.
Raw data

EspPΔ5' band intensity	S
β barrel band intensity	P
Total intensity	S+P
folding fraction	P/(S+P)

TN102-pldA	prep1	rxn2			
Sample	S	P	S+P	P/(S+P)	% folding
0.25	7165.953	0	7165.953	0	0
0.5	7474.953	0	7474.953	0	0
1	7316.539	161.263	7477.802	0.02156556	2.15655616
2.5	8633.589	291.263	8924.852	0.03263505	3.2635051
5	9296.004	465.87	9761.874	0.04772342	4.77234187
15	8842.761	586.113	9428.874	0.06216151	6.21615052
30	9757.761	868.012	10625.773	0.0816893	8.16893039

TN103-mlaA pldA	prep1	rxn2			
Sample	S	P	S+P	P/(S+P)	% folding
0.25	4727.225	0	4727.225	0	0
0.5	5949.347	0	5949.347	0	0
1	6846.054	107.778	6953.832	0.01549908	1.54990802
2.5	8971.418	329.506	9300.924	0.03542723	3.54272328
5	7828.933	463.749	8292.682	0.05592268	5.59226798
15	7429.933	593.113	8023.046	0.07392616	7.39261622
30	8315.882	798.355	9114.237	0.08759428	8.7594277

TN102-pldA	prep2	rxn1			
Sample	S	P	S+P	P/(S+P)	% folding
0.25	10392.903	0	10392.903	0	0
0.5	6781.175	0	6781.175	0	0
1	9675.246	91.364	9766.61	0.00935473	0.935473
2.5	9745.296	246.263	9991.559	0.0246471	2.46471046
5	7634.054	305.263	7939.317	0.03844953	3.84495291
15	7855.761	492.749	8348.51	0.05902239	5.90223884
30	7529.933	529.163	8059.096	0.06566034	6.56603421

TN103-mlaA pldA	prep2	rxn1			
Sample	S	P	S+P	P/(S+P)	% folding
0.25	2949.69	0	2949.69	0	0
0.5	3880.811	0	3880.811	0	0
1	4834.518	0	4834.518	0	0
2.5	6992.64	232.263	7224.903	0.03214756	3.21475596
5	8695.468	453.87	9149.338	0.04960687	4.96068677
15	9642.761	638.355	10281.116	0.06209005	6.20900494
30	9342.761	662.355	10005.116	0.06620163	6.62016312

TN102-pldA	prep2	rxn2			
Sample	S	P	S+P	P/(S+P)	% folding
0.25	2433.033	0	2433.033	0	0
0.5	3622.861	0	3622.861	0	0
1	3976.397	0	3976.397	0	0
2.5	6168.347	153.192	6321.539	0.02423334	2.42333394
5	7420.761	269.263	7690.024	0.03501459	3.50145851
15	7154.347	480.163	7634.51	0.06289375	6.28937548
30	7206.347	529.991	7736.338	0.0685067	6.85067017

TN103-mlaA pldA	prep2	rxn2			
Sample	S	P	S+P	P/(S+P)	% folding
0.25	7867.853	0	7867.853	0	0
0.5	3401.933	0	3401.933	0	0
1	3393.69	0	3393.69	0	0
2.5	5745.054	179.435	5924.489	0.030287	3.02870003
5	7823.66	382.335	8205.995	0.04659216	4.65921561
15	7875.66	538.698	8414.358	0.06402128	6.40212836
30	8671.782	643.77	9315.552	0.06910702	6.91070159

Fig. S9. The levels of model OMPs in MC4100, NR698, TN101, TN102 and TN103 at late log phase.
Uncropped blots

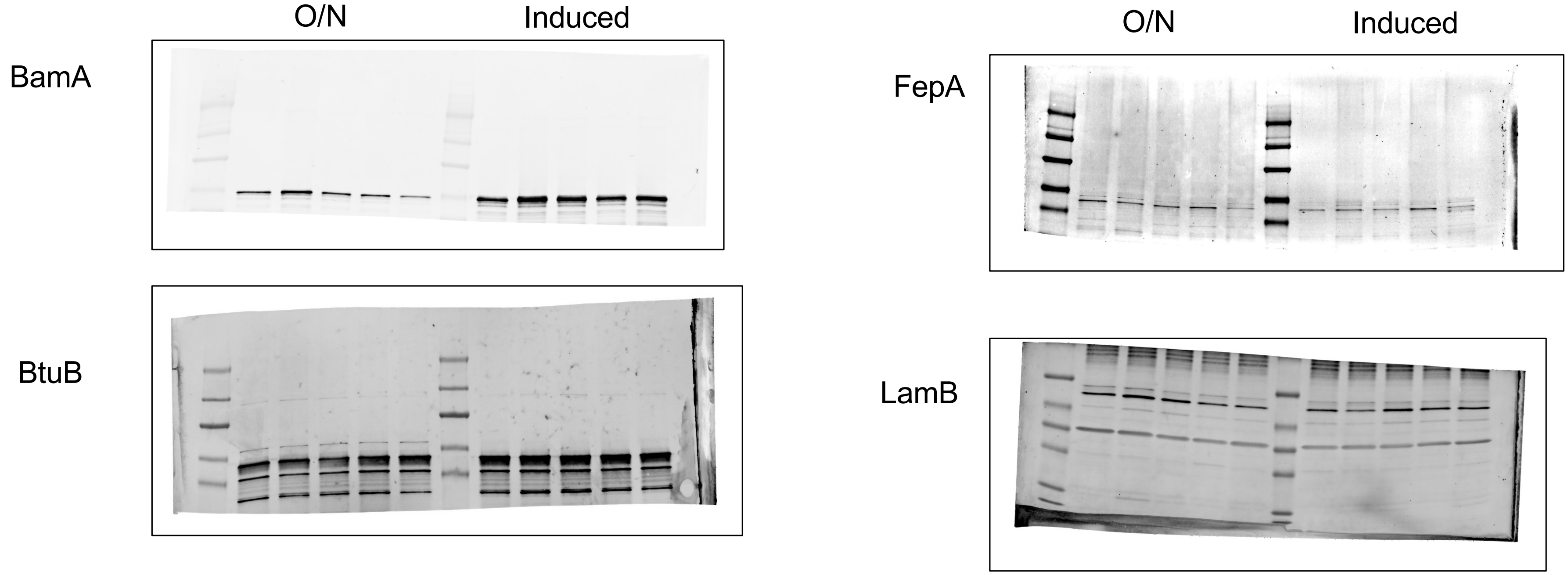

Fig. S9. The levels of model OMPs in MC4100, NR698, TN101, TN102 and TN103 at late log phase.
Uncropped blots

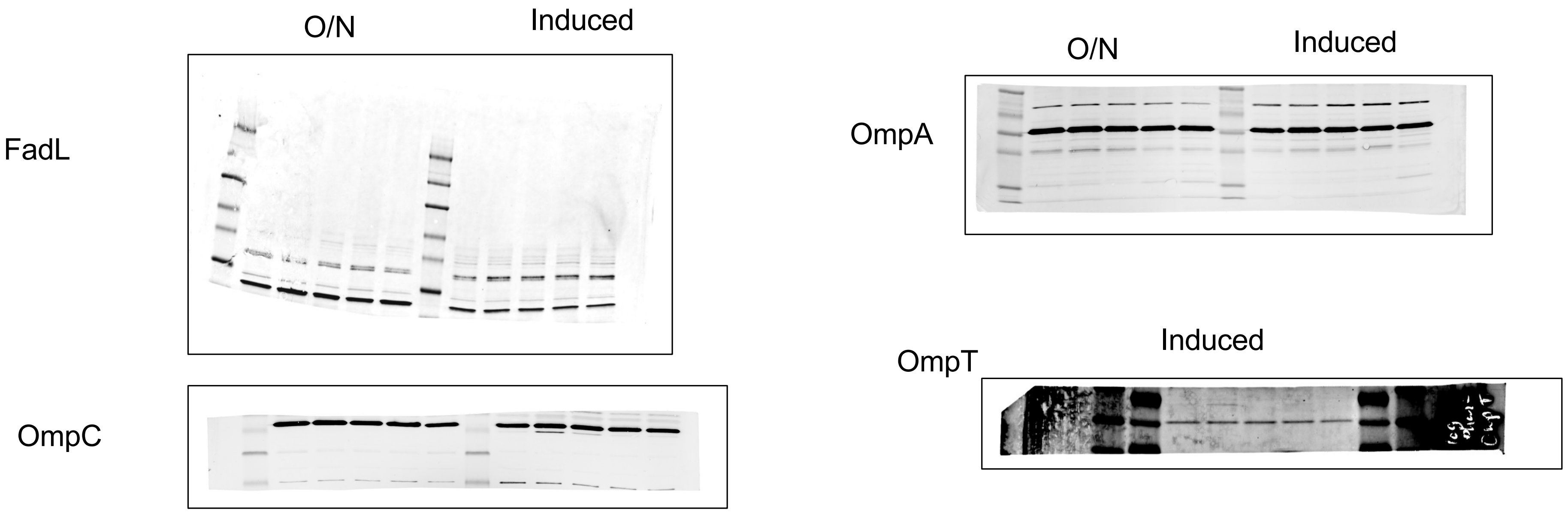

Fig. S9. The levels of model OMPs in MC4100, NR698, TN101, TN102 and TN103 at late log phase.
Western blots of the other 2 repeats used for OMP levels quantification (S9b)

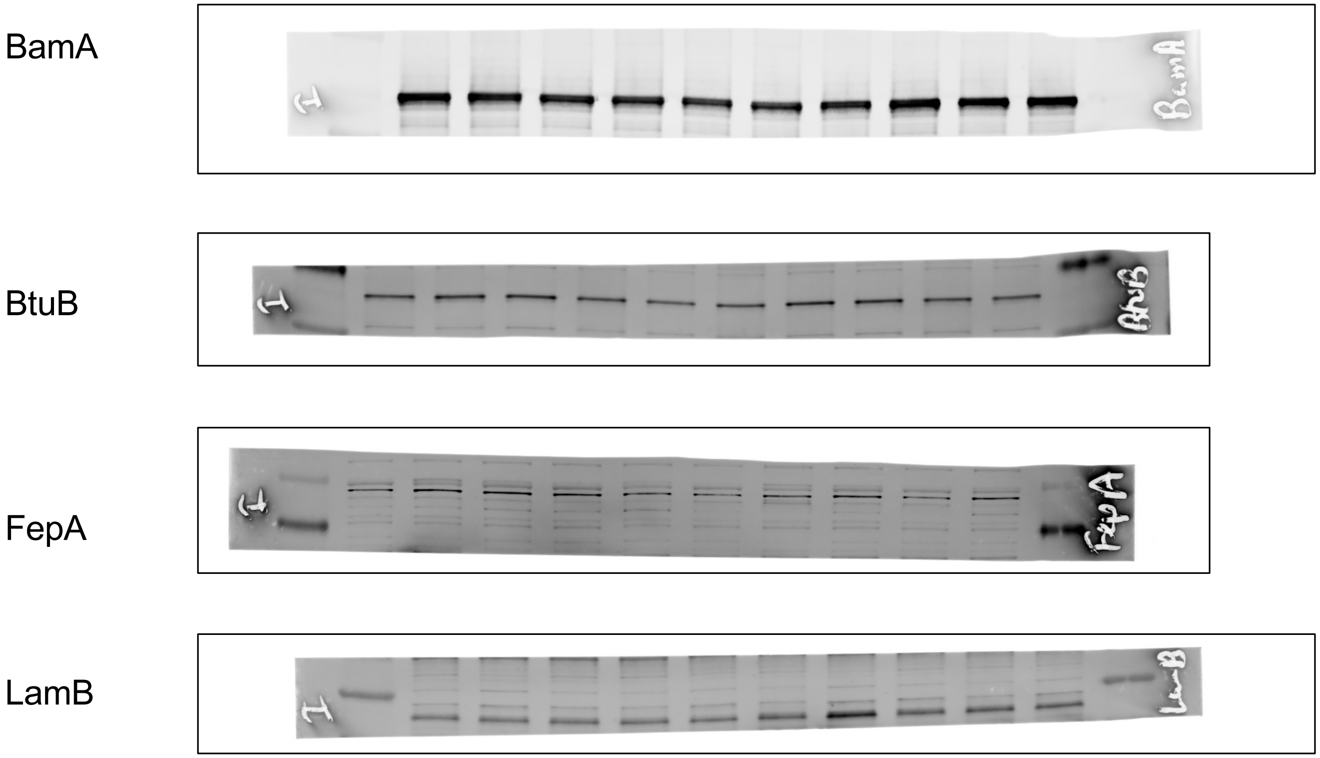

Fig. S9. The levels of model OMPs in MC4100, NR698, TN101, TN102 and TN103 at late log phase.
Western blots of the other 2 repeats used for OMP levels quantification (S9b)

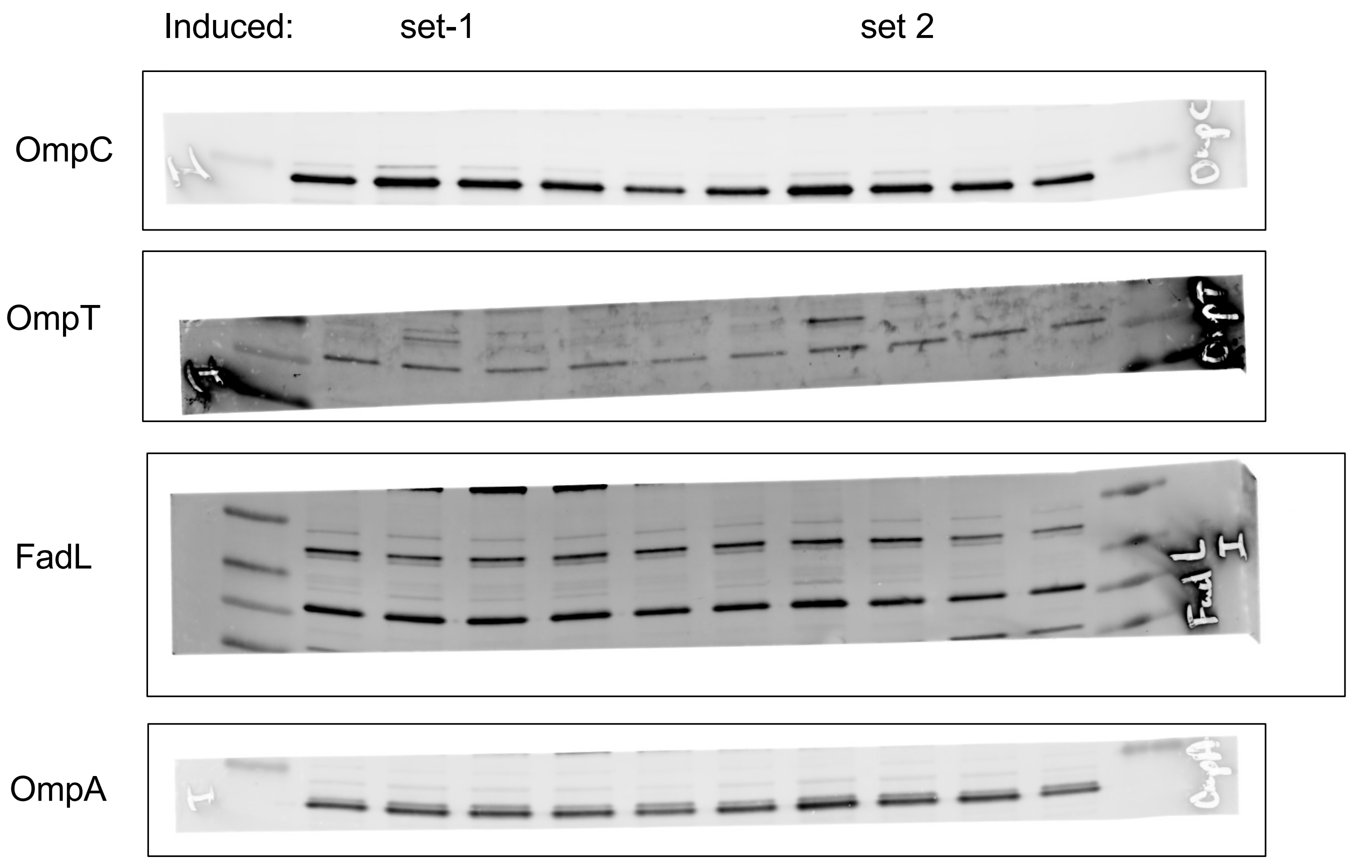

Fig. S9. The levels of model OMPs in MC4100, NR698, TN101, TN102 and TN103 at late log phase.

Signal intensities used to normalize OMPs and generate S9b (raw data, data)

OMP levels analysis in the postinduction cells

Band intensities of each OMP of the analysis

Repeat 1

WC samp	MC4100	NR698	MlaA	PldA	MP
BamA	14267.2	20279.1	17689	14305.2	16759.2
OmpC	12253.3	13077.6	13192.5	12684.9	11901.4
OmpT	8254.13	7893.3	9052.37	8079.66	6693.71
OmpA	12413.1	14263.9	13573.6	12570.5	12925.5
BtuB	7243.44	8939.2	8889.2	7218.42	7248.32
FadL	2445.08	2295.08	2475.33	2607.5	3001.1
LamB	6249.05	3636.86	7786.42	6486.18	6366.28

Repeat 2

WC samp	MC4100	NR698	MlaA	PldA	MP
BamA	22326.4	20823.2	19016.4	17420.4	14865.3
OmpC	14028.4	17053.3	15491	14281.6	9658.73
OmpT	4239.69	4087.4	2930.08	3374.28	2002.62
OmpA	10297.5	12481.2	12621.9	11341.4	10423
BtuB	3959.81	5170.42	4885.47	3774.52	3058.28
FadL	7876.93	5720.45	6433.62	7208.52	6494.15
LamB	2474.28	2760.1	2939.23	2696.52	2476.4

Repeat 3

WC samp	MC4100	NR698	MlaA	PldA	MP
BamA	15855.2	17343.5	21158.1	20327.1	21640.2
OmpC	12957.4	18113	15008.3	13918.7	11805.5
OmpT	2562.03	4671.05	3727.69	4235.4	2923.81
OmpA	11183.9	14991.3	12198.7	11501.5	8891.27
BtuB	3432.45	4698.1	4962.18	3837.81	3645.93
FadL	7130.1	7500.15	7220.86	4380.74	4394.35
LamB	3108.47	5867.95	3452.64	3237.93	2645.28

Norm with respect to WT OMP levels

Repeat 1

WC samp	MC4100	NR698	MlaA	PldA	MP
BamA	1.000	1.421	1.240	1.003	1.175
OmpC	1.000	1.067	1.077	1.035	0.971
OmpT	1.000	0.956	1.097	0.979	0.811
OmpA	1.000	1.149	1.093	1.013	1.041
BtuB	1.000	1.234	1.227	0.997	1.001
FadL	1.000	0.939	1.012	1.066	1.227
LamB	1.000	0.582	1.246	1.038	1.019

Repeat 2

WC samp	MC4100	NR698	MlaA	PldA	MP
BamA	1.000	0.933	0.852	0.780	0.666
OmpC	1.000	1.216	1.104	1.018	0.689
OmpT	1.000	0.964	0.691	0.796	0.472
OmpA	1.000	1.212	1.226	1.101	1.012
BtuB	1.000	1.306	1.234	0.953	0.772
FadL	1.000	0.726	0.817	0.915	0.824
LamB	1.000	1.116	1.188	1.090	1.001

Repeat 3

WC samp	MC4100	NR698	MlaA	PldA	MP
BamA	1.000	1.094	1.334	1.282	1.365
OmpC	1.000	1.398	1.158	1.074	0.911
OmpT	1.000	1.823	1.455	1.653	1.141
OmpA	1.000	1.340	1.091	1.028	0.795
BtuB	1.000	1.369	1.446	1.118	1.062
FadL	1.000	1.052	1.013	0.614	0.616
LamB	1.000	1.888	1.111	1.042	0.851

Fig. S11. The levels of model OMPs and LPS in WC samples and native OMs purified from MC4100, NR698, TN101, TN102 and TN103 that were used in the lipidomics analysis.
Uncropped blots

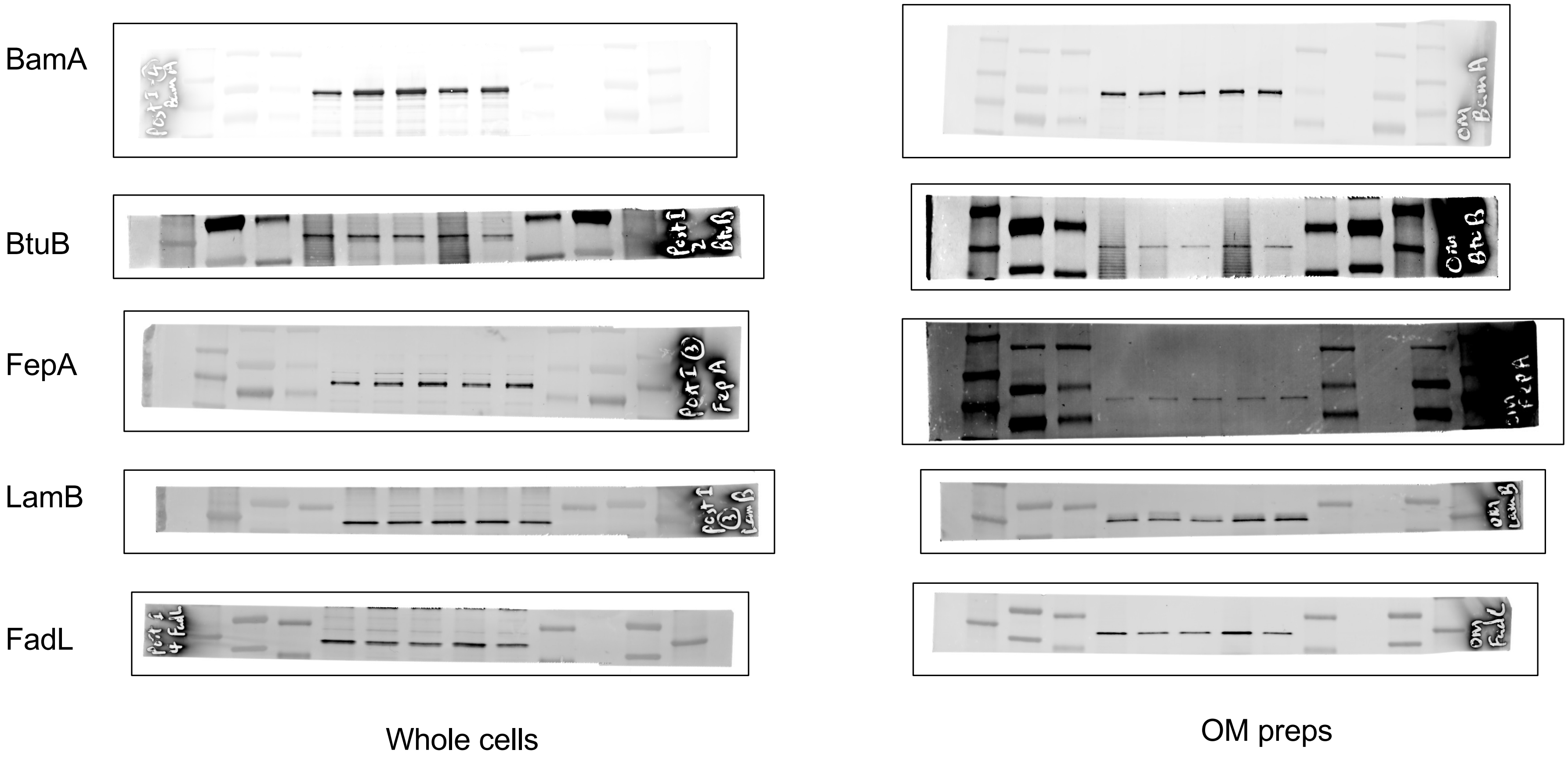

Fig. S11. The levels of model OMPs and LPS in WC samples and native OMs purified from MC4100, NR698, TN101, TN102 and TN103 that were used in the lipidomics analysis.

Uncropped blots

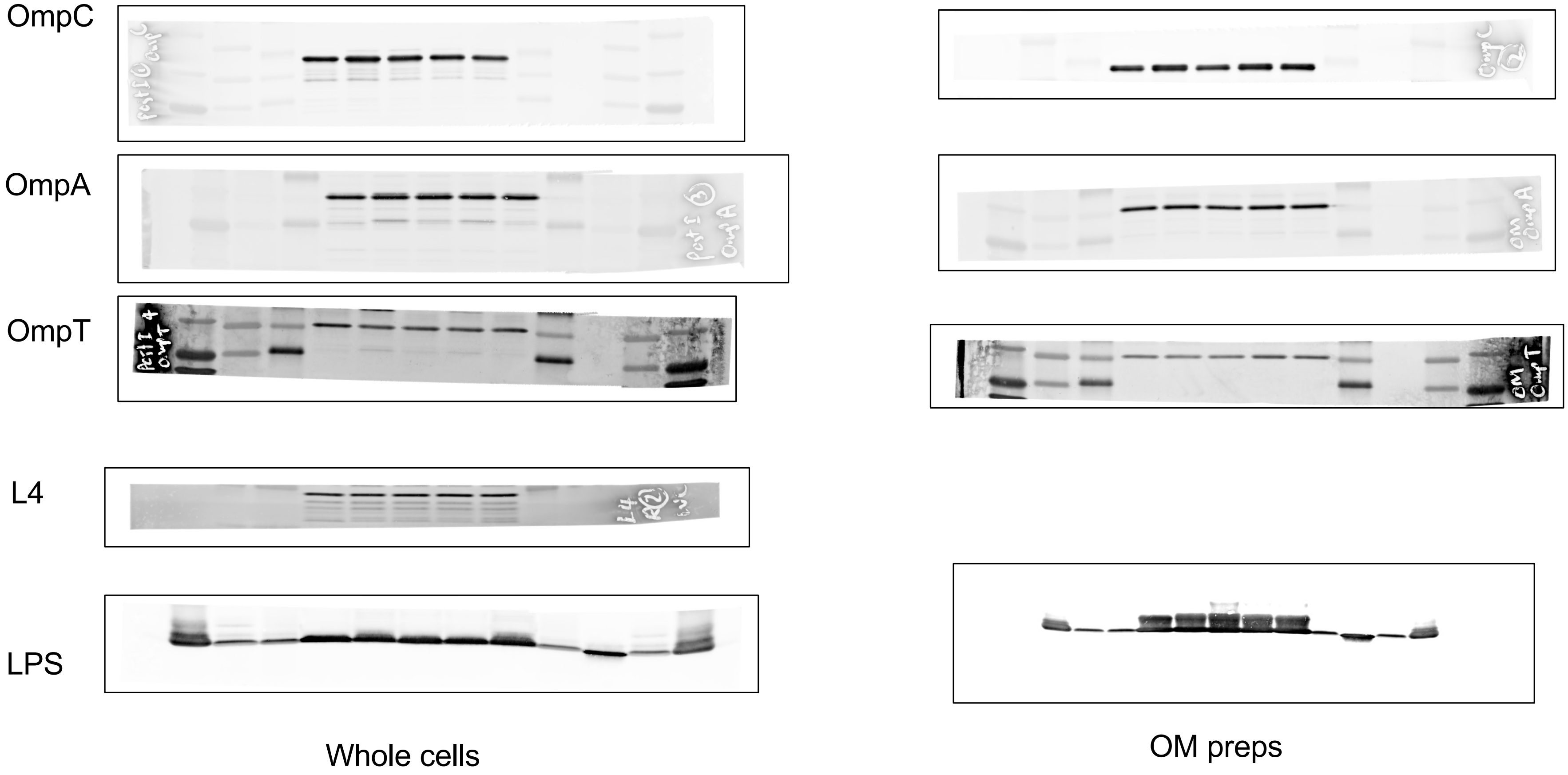

Fig. S11. The levels of model OMPs and LPS in WC samples and native OMs purified from MC4100, NR698, TN101, TN102 and TN103 that were used in the lipidomics analysis.
Western blots of the other 3 repeats used for OMP levels quantification (S11c and S11d)

BamA

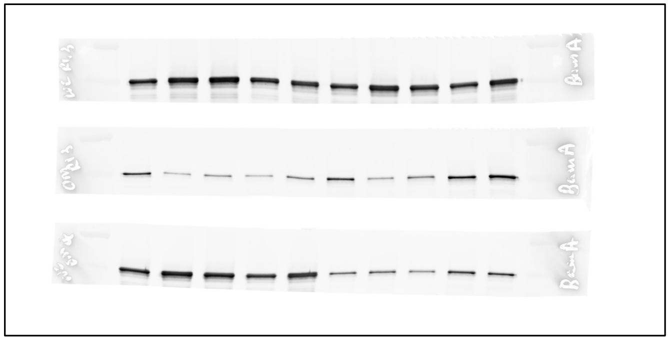

LamB

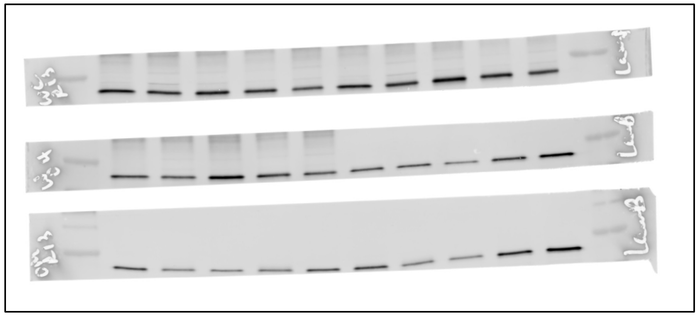

BtuB

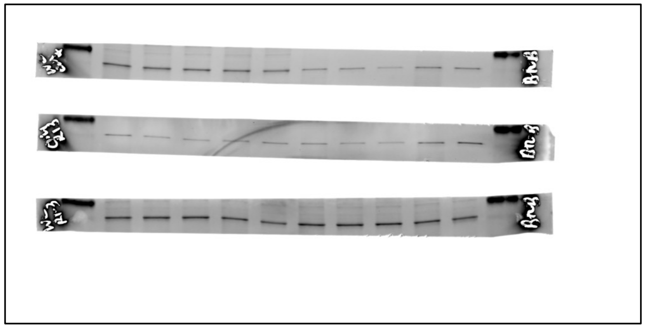

FadL

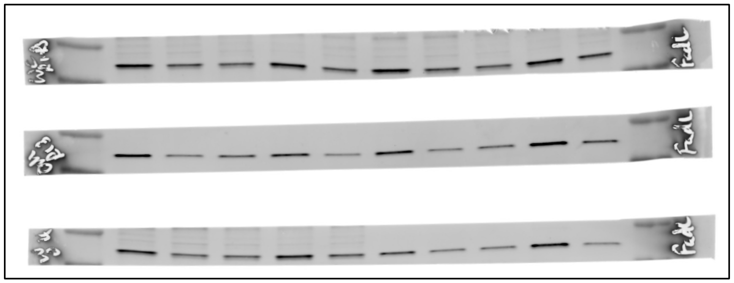

Fig. S11. The levels of model OMPs and LPS in WC samples and native OMs purified from MC4100, NR698, TN101, TN102 and TN103 that were used in the lipidomics analysis.
Western blots of the other 3 repeats used for OMP levels quantification (S11c and S11d)

OmpC

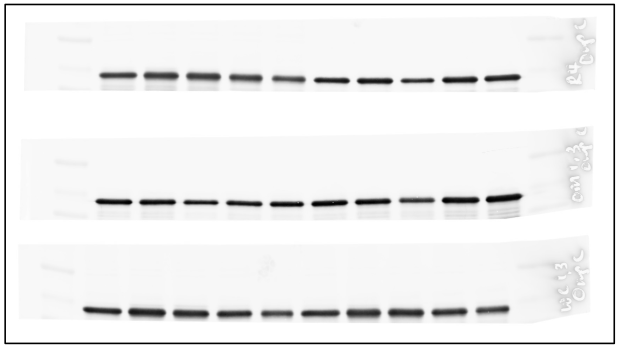

OmpT

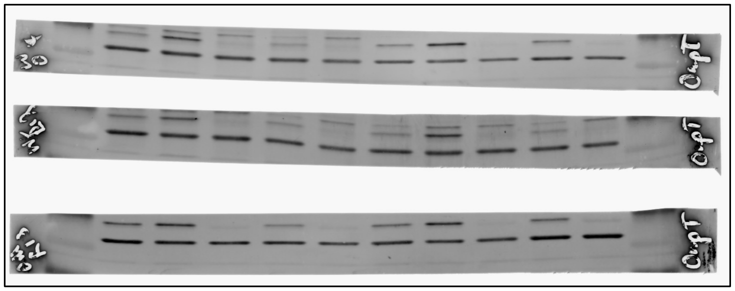

OmpA

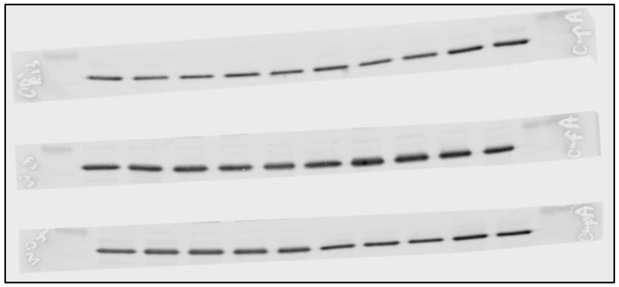

L4

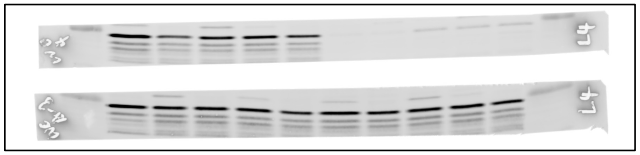

Fig. S11. The levels of model OMPs and LPS in WC samples and native OMs purified from MC4100, NR698, TN101, TN102 and TN103 that were used in the lipidomics analysis.
Western blots of the other 3 repeats used for OMP levels quantification (S11c and S11d) and LPS levels

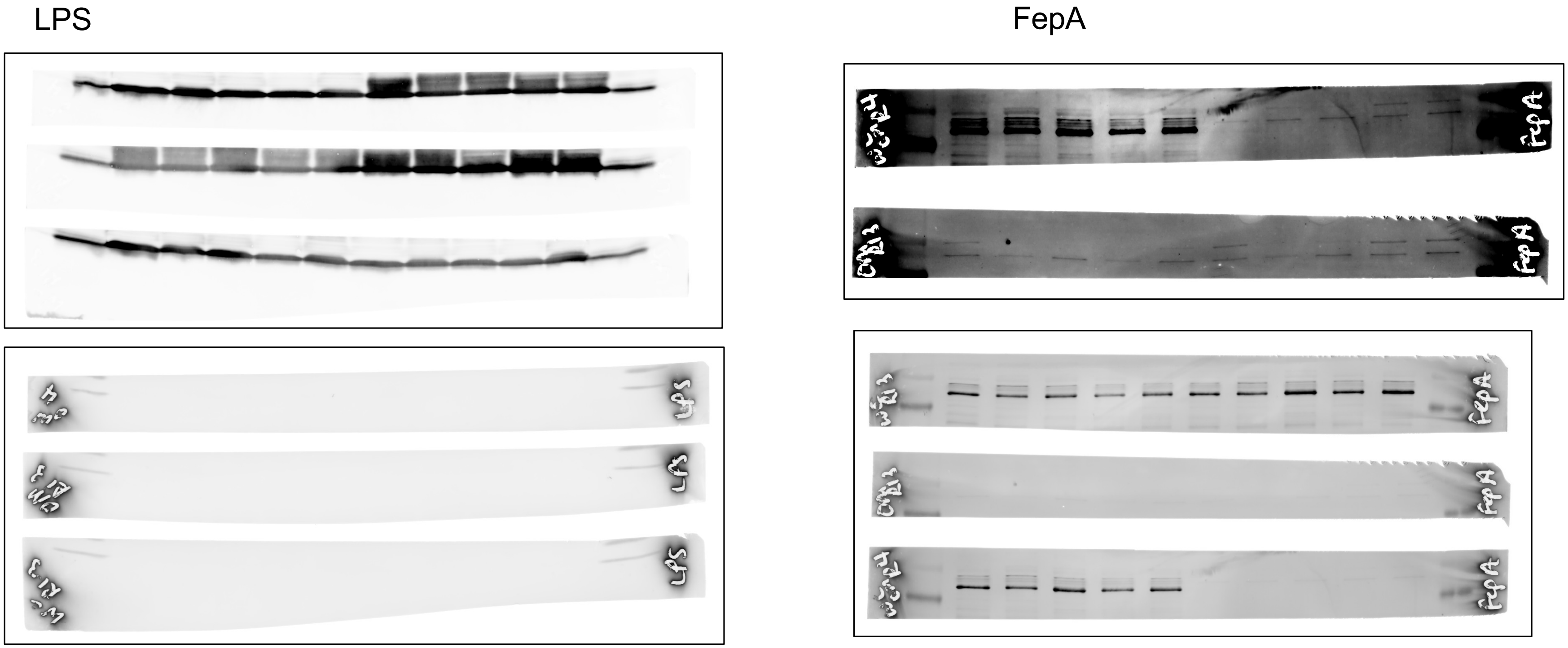

Fig. S11. The levels of model OMPs and LPS in WC samples and native OMs purified from MC4100, NR698, TN101, TN102 and TN103 that were used in the lipidomics analysis.

Signal intensities used to normalize OMPs and generate S11c, S11d

Raw data, data

OMP levels analysis in the lipidomics samples: Whole cells

Band intensities of each OMP of the analysis

Repeat 1

WC sample	MC4100	NR698	MlaA	PldA	MP
BamA	15130.1	20378.7	21198.1	15700.1	15927.3
OmpC	19623.2	25516.9	20893.6	18849.6	14935.4
OmpT	5736.3	4889.3	5164.47	4255.59	3586.64
OmpA	14792.6	15140.7	15914.7	14475.7	13669.9
BtuB	4547.83	5324.49	5694.9	4462.42	3608.08
FadL	6563.18	3912.05	3581.64	6342.25	3835.05
LamB	9423.15	7571.27	9278.9	8219.73	5853.08
L4	9593.81	7593.28	7908.86	8284.35	6035.4

Repeat 2

WC samp	MC4100	NR698	MlaA	PldA	MP
BamA	12381.6	19016.2	20167	14355.1	16078.9
OmpC	12718.9	14625	12719.7	11792	10694.6
OmpT	10772.9	8094.64	8803.88	8669.88	9459.76
OmpA	8827.23	10163.2	9928.93	9288.81	9183.93
BtuB	5683.9	7015.68	6090.78	4712.3	4502
FadL	8568.05	6455.76	5722.35	8732.83	5586.76
LamB	8030.18	6798.88	8209.83	7372.42	5550.76
L4	5297.31	5086.01	5741.55	5951.89	5740.01

Repeat 3

WC samp	MC4100	NR698	MlaA	PldA	MP
BamA	14967.6	19673.7	17386.7	14807.4	17404.8
OmpC	19782	21491.1	22040.3	19509.3	16137.7
OmpT	4608.76	4567.18	4474.3	4256.88	3756.23
OmpA	17687.8	20671.7	20419.7	18273.3	14996
BtuB	5061.83	5651.73	5503.2	5013.66	4822.15
FadL	6989.3	4191.35	3801.76	6911.25	4271.64
LamB	9185.85	8368.44	12640.5	9722.51	7160.61
L4	9336.86	7243.1	7833.88	8176.3	6589.05

Repeat 4

WC samp	MC4100	NR698	MlaA	PldA	MP
BamA	15483.5	19481.9	18230	13695.2	16939.4
OmpC	13837	15600.3	15692.2	14141.1	12005.8
OmpT	7350.83	6595	5875.88	5319.23	3945.57
OmpA	9440.15	10219	10864.4	10677.1	10315.2
BtuB	5664.37	5514.66	5011.59	4626.47	4188.71
FadL	5728.71	3271.05	3418.1	6026.64	3590.98
LamB	6092	4891	9382.49	6746.78	4973.71
L4	10808.6	4616.74	7067.45	7584.45	5454.21

Norm with respect to WT OMP levels

Repeat 1

WC sample	MC4100	NR698	MlaA	PldA	MP
BamA	1.000	1.347	1.401	1.038	1.053
OmpC	1.000	1.300	1.065	0.961	0.761
OmpT	1.000	0.852	0.900	0.742	0.625
OmpA	1.000	1.024	1.076	0.979	0.924
BtuB	1.000	1.171	1.252	0.981	0.793
FadL	1.000	0.596	0.546	0.966	0.584
LamB	1.000	0.803	0.985	0.872	0.621
L4	1.000	0.791	0.824	0.864	0.629

Repeat 2

WC samp	MC4100	NR698	MlaA	PldA	MP
BamA	1.000	1.536	1.629	1.159	1.299
OmpC	1.000	1.150	1.000	0.927	0.841
OmpT	1.000	0.751	0.817	0.805	0.878
OmpA	1.000	1.151	1.125	1.052	1.040
BtuB	1.000	1.234	1.072	0.829	0.792
FadL	1.000	0.753	0.668	1.019	0.652
LamB	1.000	0.847	1.022	0.918	0.691
L4	1.000	0.960	1.084	1.124	1.084

Repeat 3

WC samp	MC4100	NR698	MlaA	PldA	MP
BamA	1.000	1.314	1.162	0.989	1.163
OmpC	1.000	1.086	1.114	0.986	0.816
OmpT	1.000	0.991	0.971	0.924	0.815
OmpA	1.000	1.169	1.154	1.033	0.848
BtuB	1.000	1.117	1.087	0.990	0.953
FadL	1.000	0.600	0.544	0.989	0.611
LamB	1.000	0.911	1.376	1.058	0.780
L4	1.000	0.776	0.839	0.876	0.706

Repeat 4

WC samp	MC4100	NR698	MlaA	PldA	MP
BamA	1.000	1.258	1.177	0.885	1.094
OmpC	1.000	1.127	1.134	1.022	0.868
OmpT	1.000	0.897	0.799	0.724	0.537
OmpA	1.000	1.083	1.151	1.131	1.093
BtuB	1.000	0.974	0.885	0.817	0.739
FadL	1.000	0.571	0.597	1.052	0.627
LamB	1.000	0.803	1.540	1.107	0.816
L4	1.000	0.427	0.654	0.702	0.505

Fig. S11. The levels of model OMPs and LPS in WC samples and native OMs purified from MC4100, NR698, TN101, TN102 and TN103 that were used in the lipidomics analysis.

Signal intensities used to normalize OMPs and generate S11c, S11d

Raw data, data

OMP levels analysis in the lipidomics samples: OM preps

Band intensities of each OMP of the analysis

Repeat 1

OM samp	MC4100	NR698	MlaA	PldA	MP
BamA	9416.76	2427.26	4254.86	2474.84	5905.35
OmpC	16910.2	16747.5	13038.6	14906.1	15333.7
OmpT	5687.69	4573.03	3889.08	4004.08	3092.67
OmpA	7937.02	6739.25	6458.59	7899.95	7189.25
BtuB	1766.01	1125.31	908.82	1053.31	1308.6
FadL	5638.28	1961.08	2751.5	4114.62	1524.43
LamB	4968.88	2681.45	2399.5	3253.28	4212.67

Repeat 2

OM samp	MC4100	NR698	MlaA	PldA	MP
BamA	7836.93	7421.93	8250.35	9574.18	8806
OmpC	15326.4	18734.7	13711	18417.8	18946.5
OmpT	5832.4	5418.52	5558.4	7647.76	7769.76
OmpA	11897.9	12909.8	11080.4	12922.5	13583.8
BtuB	3181.01	2503.38	1867.26	4333.01	3665.86
FadL	7066.91	4045.26	4115.96	9011.62	4409.79
LamB	5496.95	5066.47	3881.05	7854.1	8245.1

Repeat 3

OM samp	MC4100	NR698	MlaA	PldA	MP
BamA	9762.71	3399.91	6373.88	12876.4	14867.6
OmpC	18152	17568.9	13304.3	20248	20325.3
OmpT	4208.15	3389.21	3861.98	5932.98	6237.98
OmpA	8582.2	6947.54	8213.2	10824.3	11222.7
BtuB	1260.94	745.82	757.527	2657.21	3276.74
FadL	5044.28	1612.72	2419.08	6442.86	3058.21
LamB	5353.86	3447.81	2857.57	7179.88	9581.88

Repeat 4

OM samp	MC4100	NR698	MlaA	PldA	MP
BamA	5551.98	6156.23	3892.62	8666.18	7892.93
OmpC	13900	15386.2	10412.1	16123.3	15327.4
OmpT	4059.26	3985.79	3369.08	5446.33	4331.33
OmpA	9073.35	9660.64	8767.81	11311.8	10764.6
BtuB	1825.13	1916.84	928.062	2407.38	2447.08
FadL	3648.03	1968.26	2264.21	6166.52	1740.26
LamB	4758.05	4518.81	2774.33	6146.59	8320.88

Norm with respect to WT OMP levels

Repeat 1

OM samp	MC4100	NR698	MlaA	PldA	MP
BamA	1.0000	0.2578	0.4518	0.2628	0.6271
OmpC	1.0000	0.9904	0.7710	0.8815	0.9068
OmpT	1.0000	0.8040	0.6838	0.7040	0.5437
OmpA	1.0000	0.8491	0.8137	0.9953	0.9058
BtuB	1.0000	0.6372	0.5146	0.5964	0.7410
FadL	1.0000	0.3478	0.4880	0.7298	0.2704
LamB	1.0000	0.5396	0.4829	0.6547	0.8478

Repeat 2

OM samp	MC4100	NR698	MlaA	PldA	MP
BamA	1.000	0.947	1.053	1.222	1.124
OmpC	1.000	1.222	0.895	1.202	1.236
OmpT	1.000	0.929	0.953	1.311	1.332
OmpA	1.000	1.085	0.931	1.086	1.142
BtuB	1.000	0.787	0.587	1.362	1.152
FadL	1.000	0.572	0.582	1.275	0.624
LamB	1.000	0.922	0.706	1.429	1.500
BamA for	1.000	1.084	1.148	1.297	1.183

Repeat 3

OM samp	MC4100	NR698	MlaA	PldA	MP
BamA	1.000	0.348	0.653	1.319	1.523
OmpC	1.000	0.968	0.733	1.115	1.120
OmpT	1.000	0.805	0.918	1.410	1.482
OmpA	1.000	0.810	0.957	1.261	1.308
BtuB	1.000	0.591	0.601	2.107	2.599
FadL	1.000	0.320	0.480	1.277	0.606
LamB	1.000	0.644	0.534	1.341	1.790

Repeat 4

OM samp	MC4100	NR698	MlaA	PldA	MP
BamA	1.000	1.109	0.701	1.561	1.422
OmpC	1.000	1.107	0.749	1.160	1.103
OmpT	1.000	0.982	0.830	1.342	1.067
OmpA	1.000	1.065	0.966	1.247	1.186
BtuB	1.000	1.050	0.508	1.319	1.341
FadL	1.000	0.540	0.621	1.690	0.477
LamB	1.000	0.950	0.583	1.292	1.749

Fig. S12. Analysis of NR698 phenotype.
Uncropped blot

B

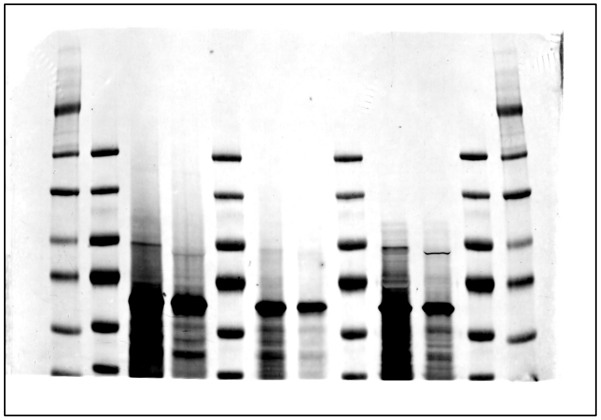

Cells grown overnight

Cells grown to OD_{600} ~0.8
